# Correction: Targeting the Intrinsically Disordered Structural Ensemble of α-Synuclein by Small Molecules as a Potential Therapeutic Strategy for Parkinson’s Disease

**DOI:** 10.1371/journal.pone.0099274

**Published:** 2014-05-27

**Authors:** The PLOS ONE Staff

## Notice of Republication

This article was republished on May 9th, 2014, due to several Supporting Information files being erroneously removed. The publisher apologizes for this error. Please view this article again for all Supporting Information files. The originally published, uncorrected article and the republished, corrected article are provided here for reference.

## Supporting Information

File S1 Originally published, uncorrected article.
(PDF)

File S2 Republished, corrected article.
(PDF)
