# Supplementary material for: Targeting the Intrinsically Disordered Structural Ensemble of α-Synuclein by Small Molecules as a Potential Therapeutic Strategy for Parkinson’s Disease
Source: PLoS One. 2014 Feb 14;9(2):e87133. doi: 10.1371/journal.pone.0087133 (PMC3925190; doi:10.1371/journal.pone.0087133)
Supplement: File S1 — (DOCX) [file pone.0087133.s001.docx]

**Supporting Information**

**Targeting the Intrinsically Disordered Structural Ensemble of α-Synuclein by Small Molecules as a Potential Therapeutic Strategy for Parkinson’s Disease**

Gergely Tóth^1,5*^, Shyra J. Gardai^5^, Wagner Zago^5^, Carlos W. Bertoncini^1,2^, Nunilo Cremades^1^, Susan L. Roy^4^, Mitali A. Tambe^4^, Jean-Christophe Rochet^4^, Celine Galvagnion^1^, Gaia Skibinski^7,8^, Steven Finkbeiner^7,8,9^, Michael Bova^5^, Karin Regnstrom^5^, San-San Chiou^5^, Jennifer Johnston^5^, Kari Callaway^5^, John P. Anderson^5^, Michael F. Jobling^5^, Alexander K. Buell^1^, Ted A. Yednock^5^, Tuomas P. J. Knowles^1^, Michele Vendruscolo^1*^, John Christodoulou^6^, Christopher M. Dobson^1*^, Dale Schenk^5*^, Lisa McConlogue^5*^

^1^Department of Chemistry, University of Cambridge, Lensfield Road, Cambridge, UK

^2^SEDIPFAR (Servicio De Descubrimiento, Diseño Y Desarrollo Pre-Clínico De Fármacos De La Argentina) drug discovery platform, Universidad Nacional de Rosario, Rosario, Argentina

^4^Department of Medicinal Chemistry and Molecular Pharmacology, Purdue University, West Lafayette, IN, USA

^5^Elan Pharmaceuticals, 700 Gateway Boulevard, South San Francisco, CA, USA

^6^Department of Structural & Molecular Biology, University College London, Gower Street, London, UK

^7^ Gladstone Institute of Neurological Disease, San Francisco, CA, USA

^8^Taube-Koret Center for Neurodegenerative Disease Research, The Consortium for Frontotemporal Dementia Research, and The Hellman Family Foundation Program for Alzheimer’s Disease Research, San Francisco, CA, USA

^9^Departments of Neurology and Physiology, UCSF, San Francisco, CA, USA

Corresponding Authors

* lisam@msg.ucsf.edu, gt293@cam.ac.uk, mv245@cam.ac.uk, dale.schenk@prothena.com, cmd44@cam.ac.uk

**Supporting Results**

***Computational identification of small molecule binders of αSyn***

From approximately 40,000 αSyn conformations generated with restrained molecular dynamics simulations using NMR derived paramagnetic relaxation enhancement distance thresholds [[1](#_ENREF_1)], 100 conformations were randomly chosen for further analysis. For the selected conformations, linear correlations were observed between the radius of gyration (R_g_), solvent accessible surface area (SASA) and the number of non-bonded contacts (Fig. 1a). Out of this set of structures, 22 conformers were randomly selected for further computational analyses. Of this subset of conformations, 18 were compact (R_g_ between 19 and 43 Å) while 4 were extended (R_g_ between 46 and 52 Å). A primary goal of this arbitrary selection process was, however, to work primarily with compact conformations as it is likely that such conformations would contain a higher number of binding pockets than extended conformations. This assumption was confirmed by the results of the computational fragment probe mapping studies (see below). The 22 selected conformations were significantly different from each other, although most of them had tertiary contacts between the NAC and C-terminal and N-terminal regions of αSyn.

To gain insights into the representative nature of the 22 conformations and the validity of the docking methodology applied in the study, two polyamines, putrescine and spermidine, previously reported to bind to the C-terminal region of the protein with low affinity [[2](#_ENREF_2)] were docked onto the structures. The docking calculations predicted that both polyamines bind well exclusively to the C-terminal region of 12 out of the 22 αSyn conformations in close proximity to residues which have been found to show significant chemical shifts changes observed by ^1^H-^15^N HSQC upon polyamine binding [[2](#_ENREF_2)], while they were predicted to bind weakly to the remaining 10 conformations at the C-terminal of the protein and/or other regions. As an example of the docking results, we illustrate the predicted highest affinity binding mode of spermidine (Fig. S1), in which it forms hydrogen bonds with side-chain hydroxyl group of Ser129, backbone carbonyl group of Ala124, and salt bridges with the side chain carboxyl groups of Asp121, Asp130, Asp135, and Glu123. The docking predictions reproduce the reported NMR results since both polyamines are predicted to interact with residues that were observed to be involved in polyamine binding by αSyn [[2](#_ENREF_2)].

**Supporting Methods**

***Materials***

The 89 compounds selected by docking calculations were purchased from different commercial vendors (Table S4) and all compounds were dissolved in pure DMSO to a final concentration of 20 mM and stored at -80 °C prior to experiments.

***Energy minimization of αSyn and small molecule conformations***

αSyn conformations [[1](#_ENREF_1)] used in the docking calculations were optimized with a steepest descent minimization procedure using the MMFF94 molecular mechanics force field [[3](#_ENREF_3),[4](#_ENREF_4)] and a distance dependent dielectric model in MOE (Computational Chemistry Group, Inc. Montreal, Canada, 2009, http://www.chemcomp.com). Low-energy conformational ensembles of the different small molecules were generated using the Systematic Conformational Search module in MOE. Rotatable bonds were systematically explored by 30 degree intervals. Each conformation generated this way was minimized using the MMFF94 force field and a distance dependent dielectric model; only those conformations were kept which had a root mean square deviation of more than 0.3 Å between their heavy atoms. AM1/BCC charges [[5](#_ENREF_5)] were used for the small molecules.

***Identification of the binding pockets***

Hot spots and binding sites were searched for using a computational fragment probe mapping methodology [[6](#_ENREF_6),[7](#_ENREF_7)]. During this process, 15 different small molecules (fragment probes) (Fig. 1b) were docked onto the whole surface of the conformations as described below in the Docking calculations section. The applied set of fragment probes contained diverse functional groups and shapes, which enabled them to bind to a variety of hot spots and binding sites. For each αSyn conformation, 100 binding solutions of each fragment probe were recorded. Hot spots were ranked according to the potential ligand efficiency (L_e_) score[[8](#_ENREF_8)] which is defined as L_e_ = ∑(E_vdW_ / number of heavy atoms contained in a fragment probe) / number of fragment probes. Hot spots were located where the strongest interacting different fragment probes clustered. They were identified when at least 4 different fragment probes bound to a pocket in the same spot and the ligand efficiency (L_e_) of each hot spot was less than -1.4 kcal/mol. These values were adopted from the results of fragment probe mapping calculations performed on the X-ray structure of JNK3 and β-secretase (Table S1). A potential binding pocket was identified when two or more high-ranking hot spots were in close proximity of each other.

***Docking calculations***

Docking calculations were carried out using FRED 2.0 (OpenEye Scientific Software, Inc., Santa Fe, www.eyesopen.com, 2008 and [[9](#_ENREF_9)]) with a translational step size of 0.5 Å and a rotational step size of 0.75 Å to exhaustively sample the low-energy conformational ensemble of the ligands within the protein binding site. The docking search was limited by checking for van der Waals clashes between the small molecule and the protein conformation (clash scale = 0.75). After the initial round of docking, the 2500 highest scoring docked poses of a small molecule were optimized within a binding pocket using the ChemGauss3 scoring function. Next, the 300 highest scoring optimized poses were further refined on the basis of consensus scoring function (Shapegauss, Chemgauss3, Oechemscore, Screenscore). First AM1/BCC charges were calculated for each ligand conformation used in the subsequent optimization step. Optimization was performed by minimizing each docked pose inside its assumed rigid protein binding site using SZYBKI 1.2.0 (OpenEye Scientific Software, Inc., www.eyesopen.com, 2008). During the SZYBKI minimization the MMFF94 molecular mechanics force field and the Poisson-Boltzmann solvation model was used. The protein structure was kept rigid while all the atoms of the ligand were allowed to be flexible.

Conformations with the lowest E_SZYBKI_ energies were considered as the prediction of the binding mode of the new molecule. E_SZYBKI_ was defined by the following equation: E_SZYBKI_ = E_intermolecular interaction_ + E_solv_ + E_ligand_, where E_intermolecular interaction_ contains all non-bonded interactions (E_vdW_ and E_Coulomb_) between the ligand and the protein, E_solv_ is the interaction energy associated with the electrostatic part of the ligand-solvent interactions, and where E_ligand_ is the potential energy of the ligand. A compound was considered to have a binding solution when E_vdw_ < 0 kcal/mol. Selection of compounds was done by (*i*) ranking compounds with binding solutions according to E_rank_ (E_rank_ = E_intermolecular interaction_ + E_solv_) and/or by ligand efficiency principles: L_Re_ = E_rank_ / number of heavy atoms in the molecule and (*ii*) the presence of complementary hydrogen bonding interactions (Fig. S2). Charged molecules were ranked separately from neutral molecules (Table S4).

***Small molecule library***

An *in silico* small molecule library of 33,000 fragment-like compounds [[10](#_ENREF_10)] was generated from 1.3 million commercially available compounds. The library was assembled by the application of a set of physico-chemical filters, which selected compounds with favorable blood-brain barrier penetration properties and high solubility.

***Transgenic animals***

Bacterial Artificial Chromosome (BAC) clone RP11-458H10, containing the human SNCA gene sequence (Life Technologies, Carlsbad, CA) was modified to generate both the Rep1 mutation [[11](#_ENREF_11)] and E46K mutation [[12](#_ENREF_12)] by BAC recombineering methods as described [[13](#_ENREF_13)]. The mutations were confirmed by sequencing. Circular BAC constructs containing the hSNCA transgene (~168 Kb) were used to perform pronuclear microinjection into B6SJL F2 mouse strains (The Jackson Laboratories, Bar Harbor, ME) in the concentration of 1-3 μg/μl followed by implantation into pseudo pregnant females (Xenogen Biosciences, Cranbury, NJ). Founders were identified by PCR genotyping using PCR primers on the 5’ end of the BAC: forward 5’-GATTTCCTTCTTTGTCTCCTATAGCACTGG-3’; reverse 5’-GAAGCAGGTATTACTGGCA GATGAGGC-3’; the middle of the BAC: forward5’-GCCCTGTTTAGCAATCAACCTTCC-3’;reverse 5’-TACCTTGGAGTGAACCCTAATCTGACTG-3’; the 3’ end of the BAC: forward 5’-GTGGTACACCGAGCAGTGGAAATGAG-3’; reverse 5’TGTCTGTTATCACCTTAAGTCTACTTTTGTCAGC-3’. Transgene copy number was determined by TaqMan qPCR method and determined as 4-5 copies. The TaqMan probe and primers for the hSNCA transgene for copy number determination were designed from 1.1 Kb exon 6 sequence (150-300 region). Probe (33 bp): FAM-CAC AAA GAC CCT GCT ACC ATG TAT TCA CTT CAG-TAMARA. Primers: forward: 5’-AGTATCTGTACCTGCCC CCACTC-3’; reverse: 5’TGAAGCCACAAAATCCACAGC-3’. Founder animals were bred with B6D2F1 mice and maintained as heterozygotes on this background with non transgenic littermates as controls. Line BAC-Tg3(SNCA^E46K^) animals were bred in sufficient numbers for a 3, 8-9, 12-14, 18-20 month old (MO) characterization cohorts and were 3-8 generations from founders. All mice were housed in a pathogen-free, climate controlled and given food and water ad libitum. All animal studies were reviewed and approved by the Institutional Animal Care and Use Committee at Elan pharmaceuticals in accordance with the National Institutes of Health Guide for the Care and Use of Laboratory Animals.

***Microglia cultures***

Microglia were obtained from cerebral cortices of neonate mice (1-3 days old). Cortices were mechanically dissociated in Hanks’ Balanced Salt Solution with 100 µg/ml DNAse I (Sigma). Dissociated cells were filtered through a 100 micron cell strainer, (Falcon, Heidelberg, Germany) and centrufuged at 200g for 5 minutes. Pellets were resuspended in growth medium consisting of high glucose DMEM (Life Technologies), 10% FBS (Atlanta Biologicals, Lawrenceville,GA), and 25 ng/ml recombinant mouse granulocyte-monocyte colony stimulating factor, (rmGM-CSF, R&D Systems, Minneapolis, MN,). Each pup was cultured and genotyped individually when derived from heterozygote by herozygote crosses or for homozygote experiments cells were plated at a density of two brains per T-75 plastic culture flask. After 7 days, the flasks were shaken at 200 r.p.m. using a Lab-Line orbital shaker for 2 hours at 37^0^ C. Cells suspensions were centrufuged at 200g and re-suspended in growth medium.

**Supporting References**

1. Dedmon MM, Lindorff-Larsen K, Christodoulou J, Vendruscolo M, Dobson CM (2005) Mapping long-range interactions in alpha-synuclein using spin-label NMR and ensemble molecular dynamics simulations. Journal of the American Chemical Society 127: 476-477.

2. Fernandez CO, Hoyer W, Zweckstetter M, Jares-Erijman EA, Subramaniam V, et al. (2004) NMR of alpha-synuclein-polyamine complexes elucidates the mechanism and kinetics of induced aggregation. The EMBO journal 23: 2039-2046.

3. Halgren TA (1996) Merk Molecular Force Field: I-V. J Comp Chem 17: 490-519.

4. Halgren TA (1999) Merk Molecular Force Field: V-VII. J Comp Chem 20: 720-729.

5. Jakalian A, Jack DB, Bayly CI (2002) Fast, efficient generation of high-quality atomic charges. AM1-BCC model: II. Parameterization and validation. Journal of computational chemistry 23: 1623-1641.

6. Kortvelyesi T, Dennis S, Silberstein M, Brown L, 3rd, Vajda S (2003) Algorithms for computational solvent mapping of proteins. Proteins 51: 340-351.

7. Landon MR, Lancia DR, Jr., Yu J, Thiel SC, Vajda S (2007) Identification of hot spots within druggable binding regions by computational solvent mapping of proteins. Journal of medicinal chemistry 50: 1231-1240.

8. Zhu M, De Simone A, Schenk D, Toth G, Dobson CM, et al. (2013) Identification of small-molecule binding pockets in the soluble monomeric form of the Abeta 42 peptide. The Journal of Chemical Physics 139: 035101.

9. McGann M (2011) FRED pose prediction and virtual screening accuracy. Journal of chemical information and modeling 51: 578-596.

10. Congreve M, Carr R, Murray C, Jhoti H (2003) A 'rule of three' for fragment-based lead discovery? Drug discovery today 8: 876-877.

11. Farrer M, Maraganore DM, Lockhart P, Singleton A, Lesnick TG, et al. (2001) alpha-Synuclein gene haplotypes are associated with Parkinson's disease. Human molecular genetics 10: 1847-1851.

12. Zarranz JJ, Alegre J, Gomez-Esteban JC, Lezcano E, Ros R, et al. (2004) The new mutation, E46K, of alpha-synuclein causes Parkinson and Lewy body dementia. Annals of neurology 55: 164-173.

13. Liu Y, Lashuel HA, Choi S, Xing X, Case A, et al. (2003) Discovery of inhibitors that elucidate the role of UCH-L1 activity in the H1299 lung cancer cell line. Chem Biol 10: 837-846.

**Supporting Tables**

**Table S1.**  Hot spots identified in binding pockets of JNK3 kinase and β-secretase.

| Structures^a^ | Hot spot^b^ | Probes^c^ | L_e_ ^d^ |
| --- | --- | --- | --- |
| JNK3 kinase active site (ATP binding site)^e^ | | |  |
| 1 | Selectivity site | 10 | -1.45 |
| 1 | K25,F62,L55 | 11 | -1.57 |
| 1 | Y210,Y239,L178 | 7 | -1.85 |
| 1 | ATP hinge | 13 | -1.49 |
|  |  |  |  |
| JNK3 kinase DFG-out site^f^ | |  |  |
| 2 | DFG-out “phenyl” site | 4 | -2.17 |
| 2 | DFG-out “top” site | 14 | -2.00 |
| 2 | Selectivity site | 4 | -1.68 |
| 2 | K25,F62,L55 | 5 | -1.87 |
|  |  |  |  |
| β-secratse substrate binding site | |  |  |
| 3 | Y17, V16 A15 | 14 | -1.93 |
| 3 | V31, E34 V33 | 10 | -1.82 |

^a^protein structures determined by X-ray crystallography (in house structures from Elan Pharmaceuticals)

^b^examples of residues aligning hot spots or description of hot spot

^c^fragment probes used for computational fragment probe docking

^d^potential ligand efficiency [[8](#_ENREF_8)] of hot spot in kcal/mol

^e^within the JNK3 kinase DFG-out site, both the *selectivity site* and the *DFG-out “phenyl” site* resulted in the binding of only 4 different fragment probes.

^f^in case of the JNK3 kinse active site, the fragment probes binding to the *selectivity site* had a L_e_ of -1.45 kcal/mol.

**Table S2.** Hot spots and binding pockets identified on the 22 selected αSyn conformations.

| αSyn^a^ | Hot spot^b^ | Probes^c^ | L_e_^d^ | Pocket^e^ |
| --- | --- | --- | --- | --- |
| 1 | H50,T54,E61 | 15 | -1.45 |  |
| 1 | Y136,V118,E126 | 11 | -1.22 |  |
| 1 | Q109,P108,P117 | 6 | -1.32 |  |
|  |  |  |  |  |
| 2 | Y39,T92,V49* | 7 | -1.53 | I |
| 2 | Y39,F94,K43* | 9 | -1.19 | I |
| 2 | Y39,A91,V48* | 5 | -1.47 | I |
| 2 | E131,V48,V49 | 7 | -1.64 |  |
|  |  |  |  |  |
| 3 | E104,L38,Q24* | 12 | -1.57 | II |
| 3 | F94,V40,Q109* | 9 | -1.29 | II |
| 3 | L38,E28,Q24* | 11 | -1.73 | II |
|  |  |  |  |  |
| 4 | A124,P128,E131* | 10 | -1.35 |  |
| 4 | Y125,E126,P138* | 12 | -1.28 |  |
| 4 | K80,E46,T65* | 12 | -1.22 |  |
| 4 | H50,E61,V63* | 5 | -1.57 |  |
|  |  |  |  |  |
| 5 | K60,E83,V48* | 12 | -1.46 |  |
| 5 | T33,E61,G47* | 6 | -1.29 |  |
| 5 | V118,V82,S87* | 8 | -1.43 |  |
|  |  |  |  |  |
| 6 | Y125,E130,Q134 | 15 | -1.56 |  |
| 6 | V40,V52,K34 | 15 | -1.70 |  |
|  |  |  |  |  |
| 7 | Y133,S129,D135* | 15 | -1.64 | III |
| 7 | Q134,E130,D135* | 8 | -1.51 | III |
|  |  |  |  |  |
| 8 | V70,L113,E104* | 9 | -1.18 |  |
| 8 | F4,K6,M1 | 7 | -1.57 |  |
|  |  |  |  |  |
| 9 | V70,E61,A56* | 5 | -1.12 |  |
| 9 | H50,T54,V70* | 14 | -1.59 |  |
|  |  |  |  |  |
| 10 | Q109,E105,N103 | 12 | -1.48 |  |
| 10 | Y133,T54,E137 | 6 | -1.76 |  |
| 10 | H50,Y39,Q62 | 6 | -1.63 |  |
|  |  |  |  |  |
| 11 | I88,E114,P128* | 15 | -2.04 |  |
| 11 | Y39,V49,K43 | 14 | -1.24 |  |
|  |  |  |  |  |
| 12 | K102,P108,E105 | 11 | -1.31 |  |
| 12 | V74,T64,G67 | 10 | -1.29 |  |
| 12 | E13,I88,S87 | 5 | -1.52 |  |
| 12 | Y125,Y133,S129 | 7 | -1.34 |  |
|  |  |  |  |  |
| 13 | K43,K6,K32* | 15 | -1.66 | IV |
| 13 | V49,K43,G51* | 12 | -1.48 | IV |
| 13 | Q99,P108,E104 | 15 | -1.36 |  |
|  |  |  |  |  |
| 14 | Y136,K45,V118* | 15 | -1.76 | V |
| 14 | V49,Q109,T44* | 5 | -1.66 | V |
|  |  |  |  |  |
| 15 | Y136,V49,K43 | 14 | -1.59 |  |
| 15 | Y39,H50,V118 | 12 | -1.40 |  |
| αSyn^a^ | Hot spot^b^ | Probes^c^ | L_e_^d^ | Pocket^e^ |
|  |  |  |  |  |
| 16 | P108,V37,K17* | 14 | -1.78 | VI |
| 16 | K34,L111,E110* | 9 | -1.66 | VI |
|  |  |  |  |  |
| 17 | K32,A18,Q24* | 14 | -1.43 | VII |
| 17 | Y133,V52,T54* | 13 | -1.49 | VII |
| 17 | P128,T44,A124* | 12 | -1.55 |  |
|  |  |  |  |  |
| 18 | K45,Y125,N124* | 6 | -1.31 | VIII |
| 18 | E46,K80,V77* | 14 | -1.59 | VIII |
| 18 | Y136,E126,A124* | 13 | -1.47 | VIII |
| 18 | E46,Y125,A124* | 9 | -1.45 | VIII |
| 18 | L100,E46, Y125* | 7 | -1.07 | VIII |
|  |  |  |  |  |
| 19 | V63,T72,N65* | 6 | -1.43 |  |
|  |  |  |  |  |
| 20 | A107,E110,L113* | 7 | -1.685 |  |
|  |  |  |  |  |
| 21 | E137,E131,E130* | 12 | -1.18 |  |
|  |  |  |  |  |
| 22 | K21,A53,E20* | 15 | -0.60 |  |

^a^αSyn conformation from NMR ensemble

^b^examples of residues aligning hot spots

^c^number of fragment probes binding to hot spot

^d^potential ligand efficiency [[8](#_ENREF_8)] of hot spot in kcal/mol

^e^binding pocket identified on the basis of highest ligand efficient hot spots which are in proximity of each other.

**Table S3.** Number of non-bonded residue contacts (>4.5 Å) between regions of αSyn. Contacting residues were at least five residues apart.

| αSyn conf. | NAC–N-term | NAC–C-term | N-term- C-term |
| --- | --- | --- | --- |
| 2 | 6 | 0 | 7 |
| 3 | 5 | 0 | 6 |
| 7 | 3 | 10 | 1 |
| 13 | 0 | 0 | 0 |
| 14 | 0 | 2 | 37 |
| 16 | 41 | 10 | 12 |
| 17 | 0 | 0 | 22 |
| 18 | 26 | 10 | 13 |

**Table S4.** 89 compounds corresponding to *in silico* hits identified from results of the docking runs for each Pocket on the basis of E_rank_ and/or by L_Re_. The compounds were purchased from chemical vendors and their integrity and purity analyzed by LC/MS. Compound 7 (ELN484228) was tested in a battery of αSyn assays. Compound 38 (ELN484217) was used as a control in the phagocytosis assay.


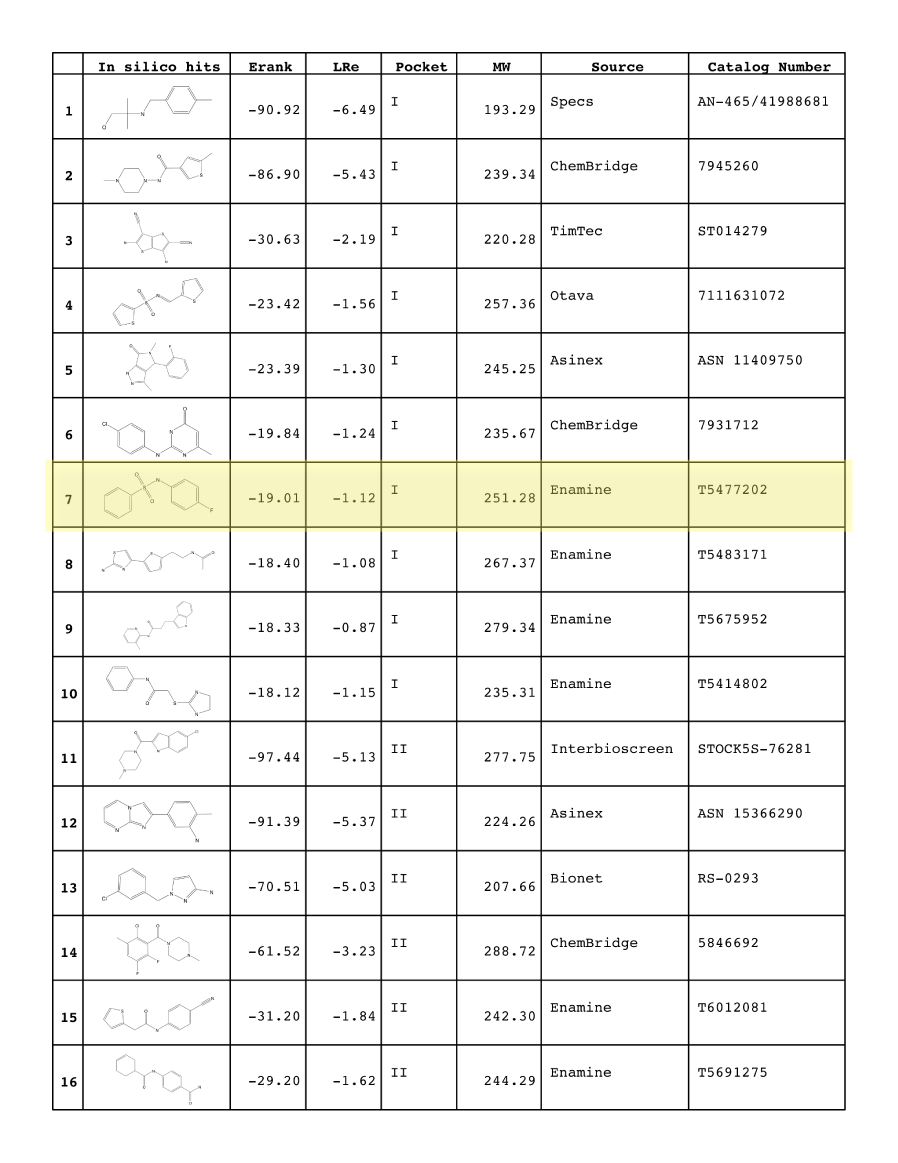


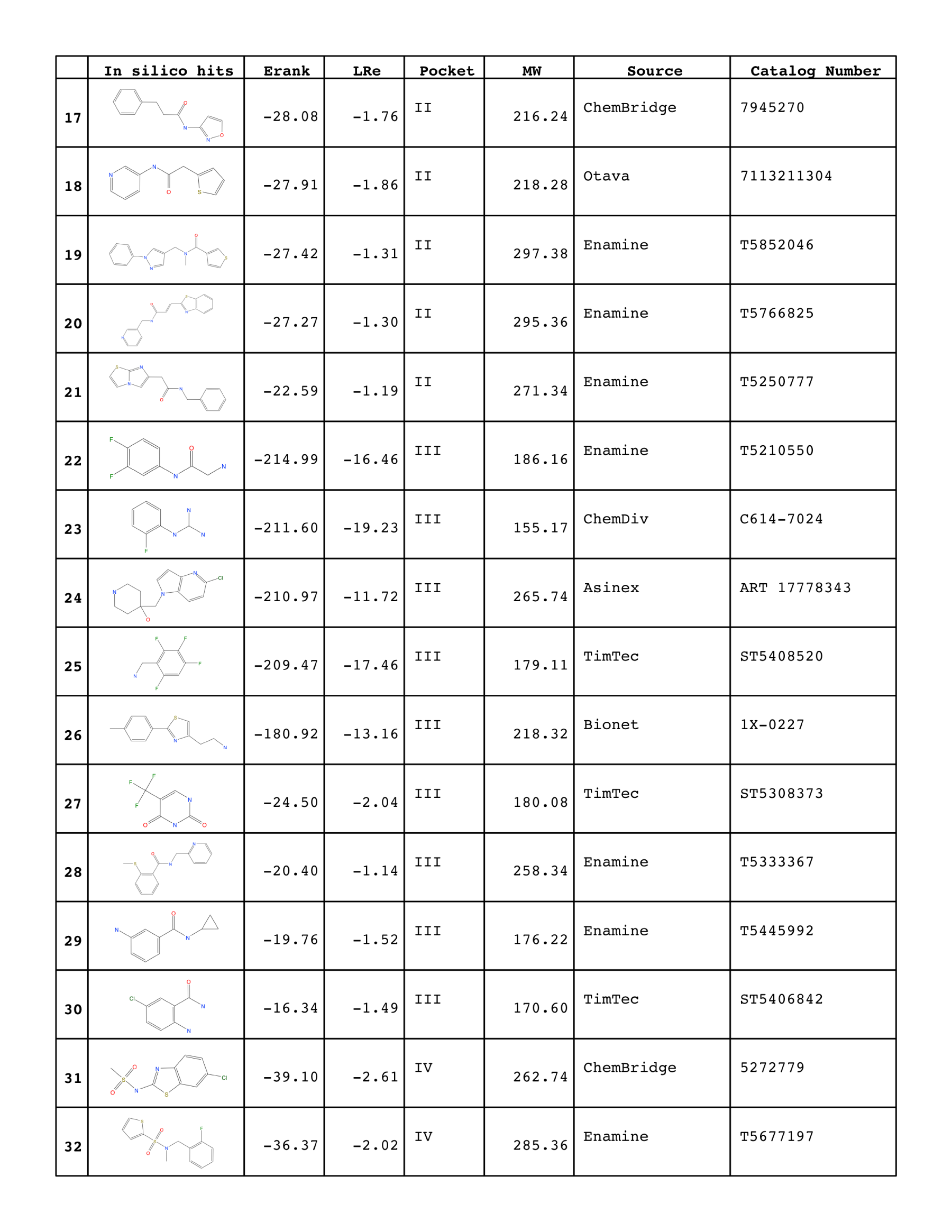

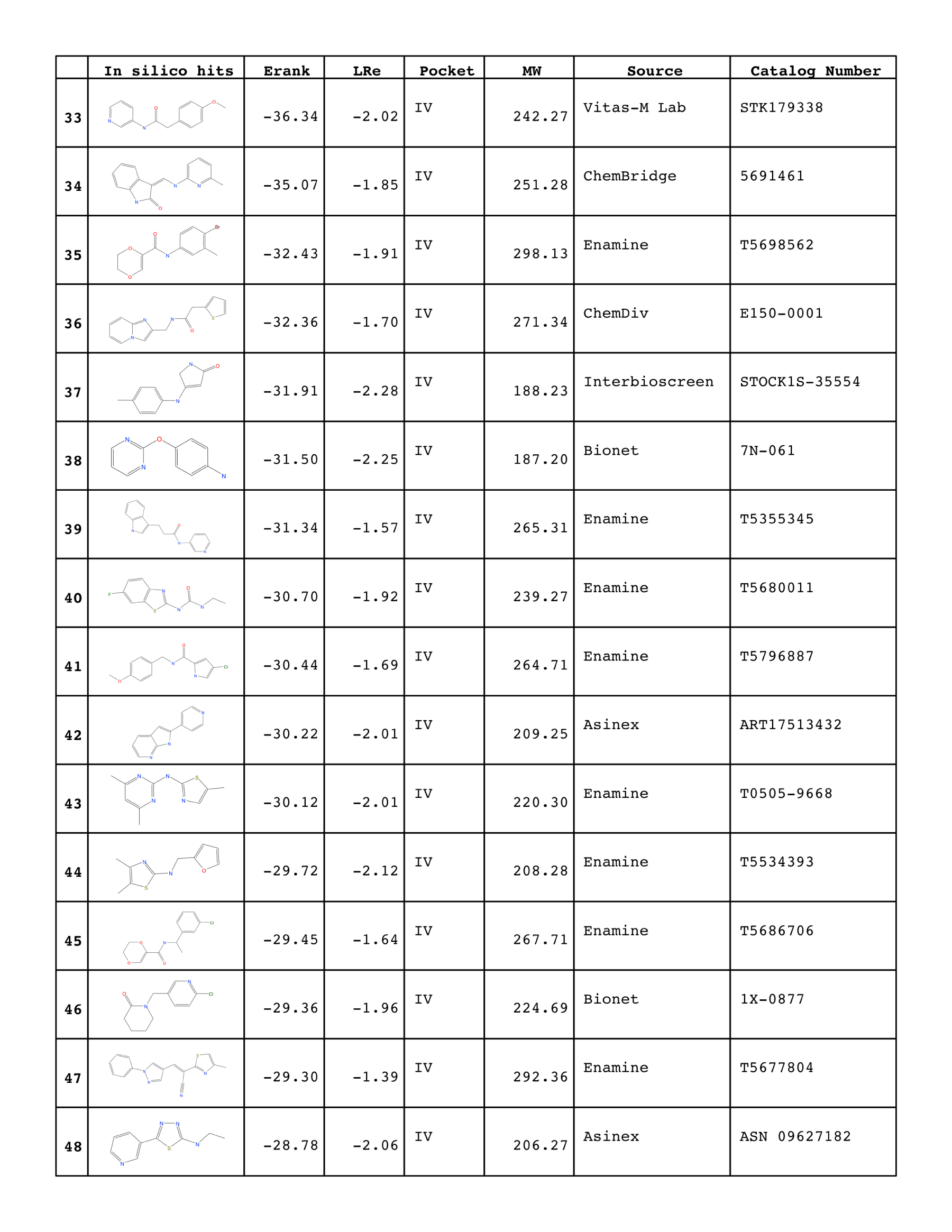

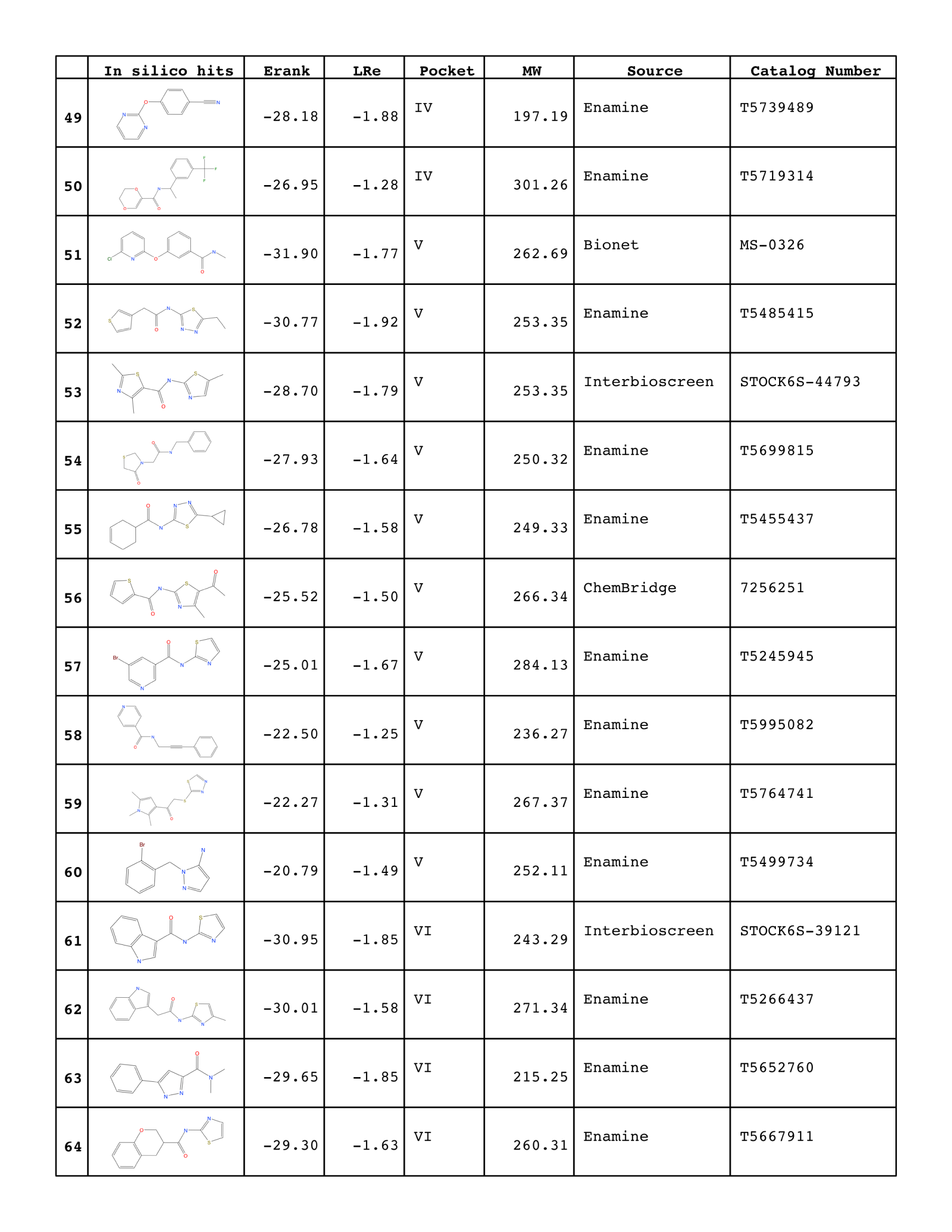

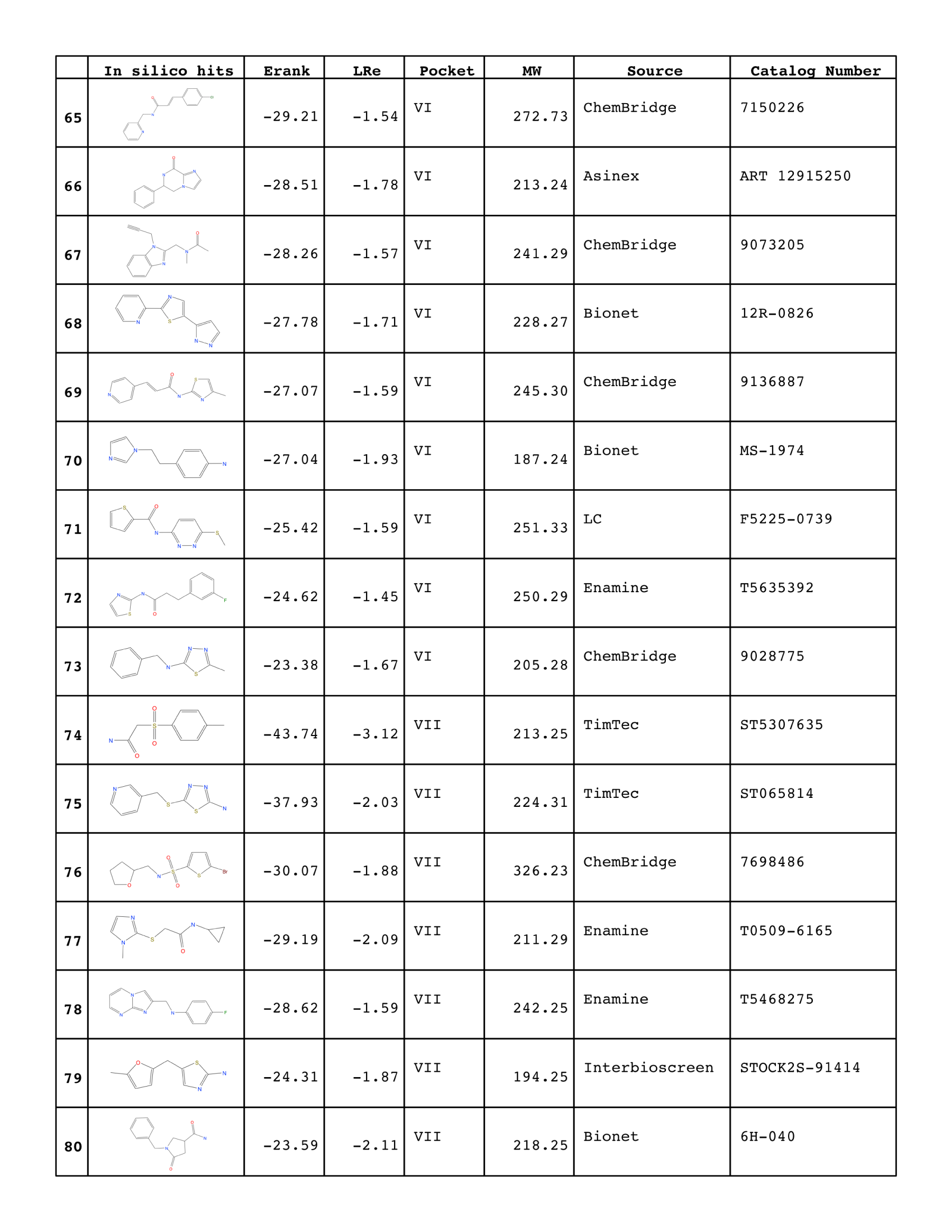

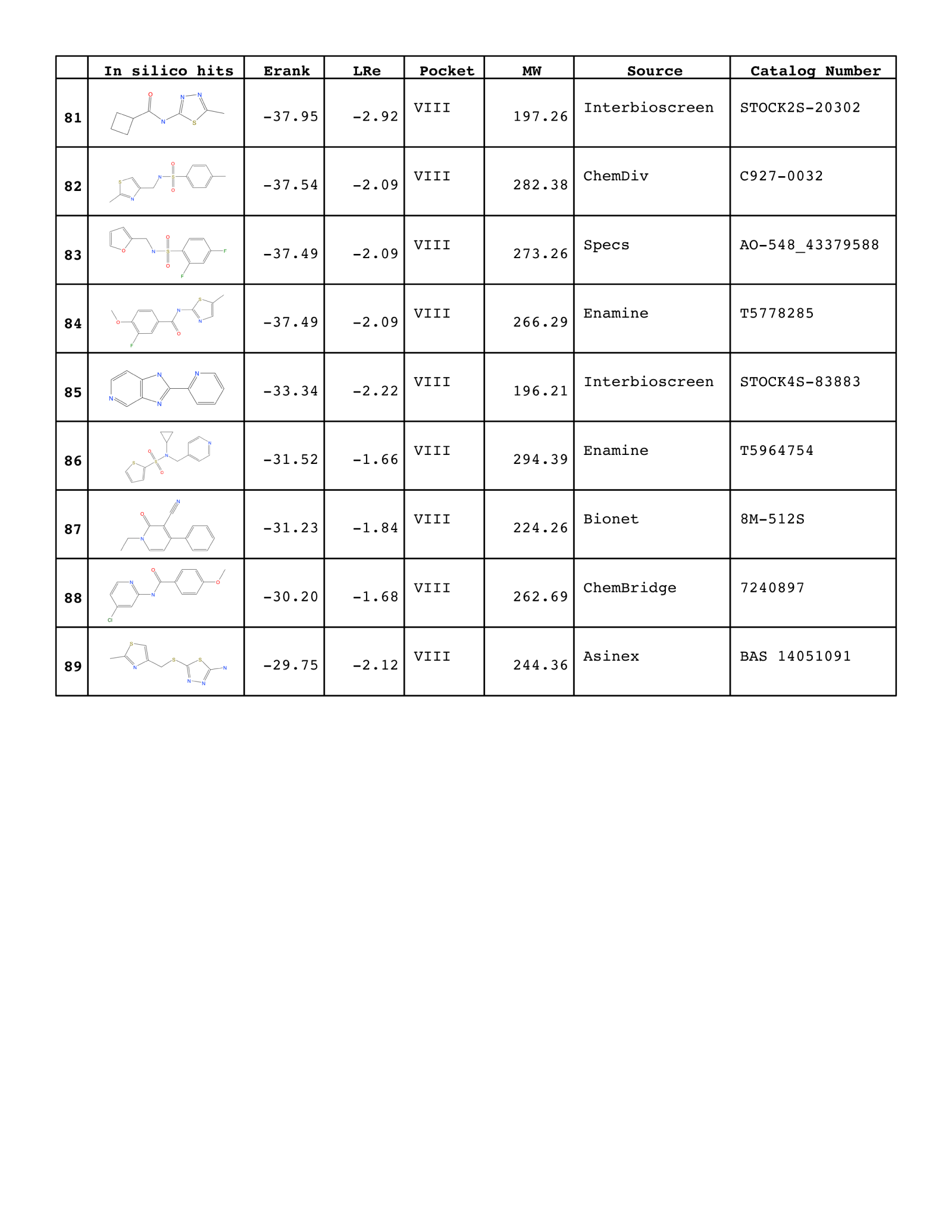


Neutral molecules were ranked separately from charged molecules.

E_rank_ = E_intermolecular interaction_ + E_solv_

L_Re_ = E_rank_ / number of heavy atoms in molecule

MW = molecular weight

Source: chemical vendor company from where compounds were ordered.

The hit identified in this study (ELN484228) is highlighted in yellow (the seventh best binder according to the *in silico* screening).
