# Supplementary figures and images for: Targeting the Intrinsically Disordered Structural Ensemble of α-Synuclein by Small Molecules as a Potential Therapeutic Strategy for Parkinson’s Disease
Source: PLoS One. 2014 Feb 14;9(2):e87133. doi: 10.1371/journal.pone.0087133 (PMC3925190; doi:10.1371/journal.pone.0087133)

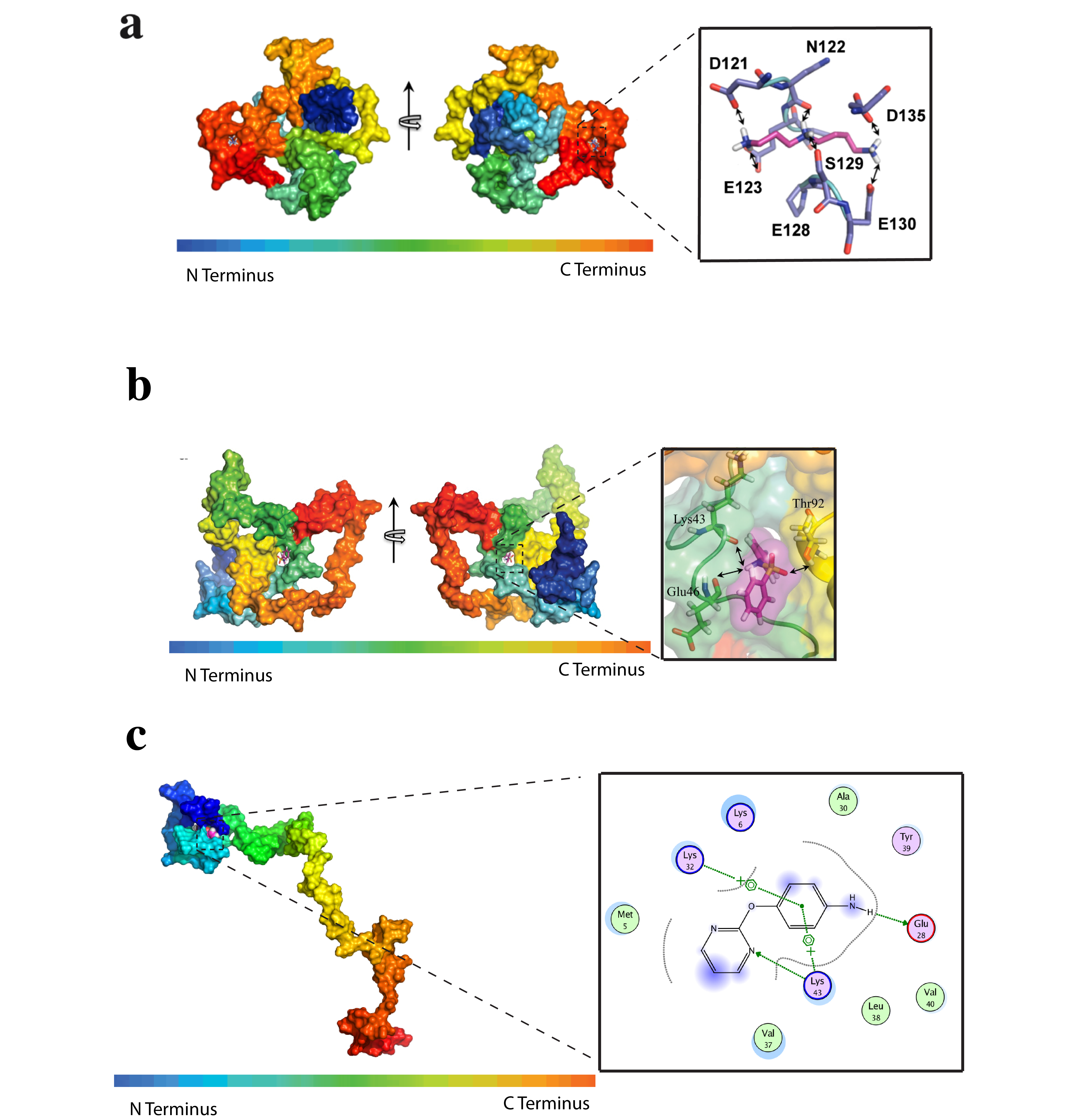

Supplement: Figure S1 — 3D models of αSyn- ligand interaction from the docking calculations . a) A representative 3D model of αSyn-spermidine interaction based on the docking calculations. Left panel: Entire surface representation of αSyn conformation 2; right panel spermidine binding pocket in αSyn conformation 2. Spermidine appears as magenta sticks and αSyn as surface representation or blue sticks. Black arrow illustrates a potential hydrogen bond or a salt bridge. Due to the high flexibility of spermidine and to the predominance of negatively charged side chains of the C-terminal of aSyn, spermidine has the potential to bind to many conformations of αSyn by forming charge-charge interactions. (b) A representative 3D model of αSyn-ELN484228 interaction based on the docking calculations. Left panel: surface representation of Conformation 2 of αSyn in complex with ELN484228. Right panel: close-up view of ELN484228 binding to Pocket I of Conformation 2. Black arrow illustrates a potential hydrogen bond or a salt bridge. (c) A representative model of the αSyn-ELN484217 interaction based on the docking calculations. ELN484217 is predicted to bind to the N-terminal portion of αSyn conformation 13. Left panel: 3D surface representation of conformation 13 of αSyn in complex with ELN484217. Right panel: close-up view of ELN484217 binding to the N-terminal of αSyn conformation 13 depicted in a representative 2D model of αSyn-ELN484217 showing hydrogen bonds (green arrow) and cation-aromatic interactions (green arrow with aromatic+). Residues of αSyn in the surface representation are coloured accordingly their location in primary sequence as shown at the bottom of panels on the left hand side. (TIF) [file pone.0087133.s002.tif]

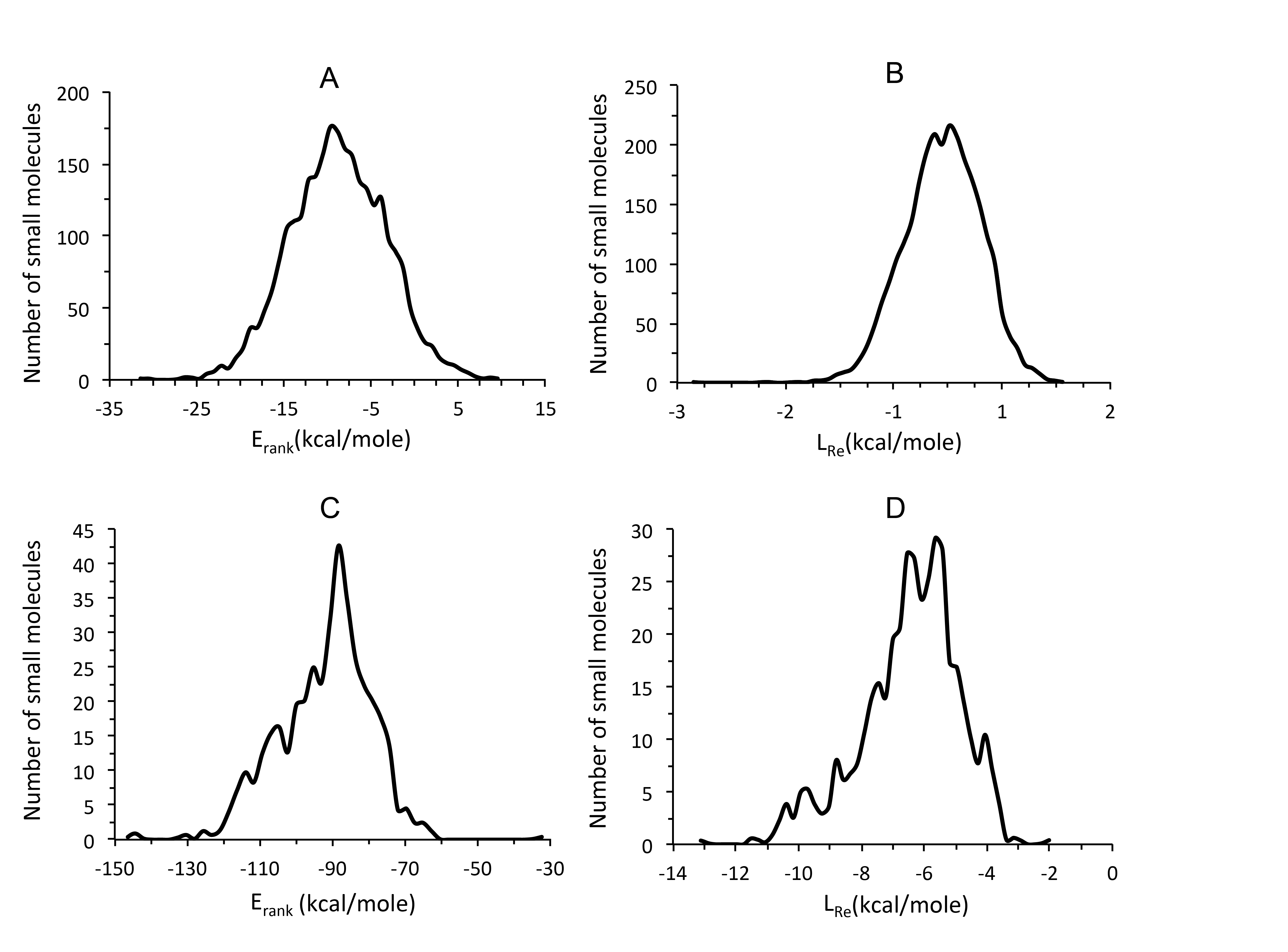

Supplement: Figure S2 — Typical profile of the results obtained after docking to a pocket in αSyn conformations. Erank distribution of neutral (a) and charged (c) small molecule binders to Pocket I of Conformation 2 of αSyn. LRe distribution of neutral (b) and charged (d) small molecule binders to Pocket I of Conformation 2 of αSyn. (TIF) [file pone.0087133.s003.tif]

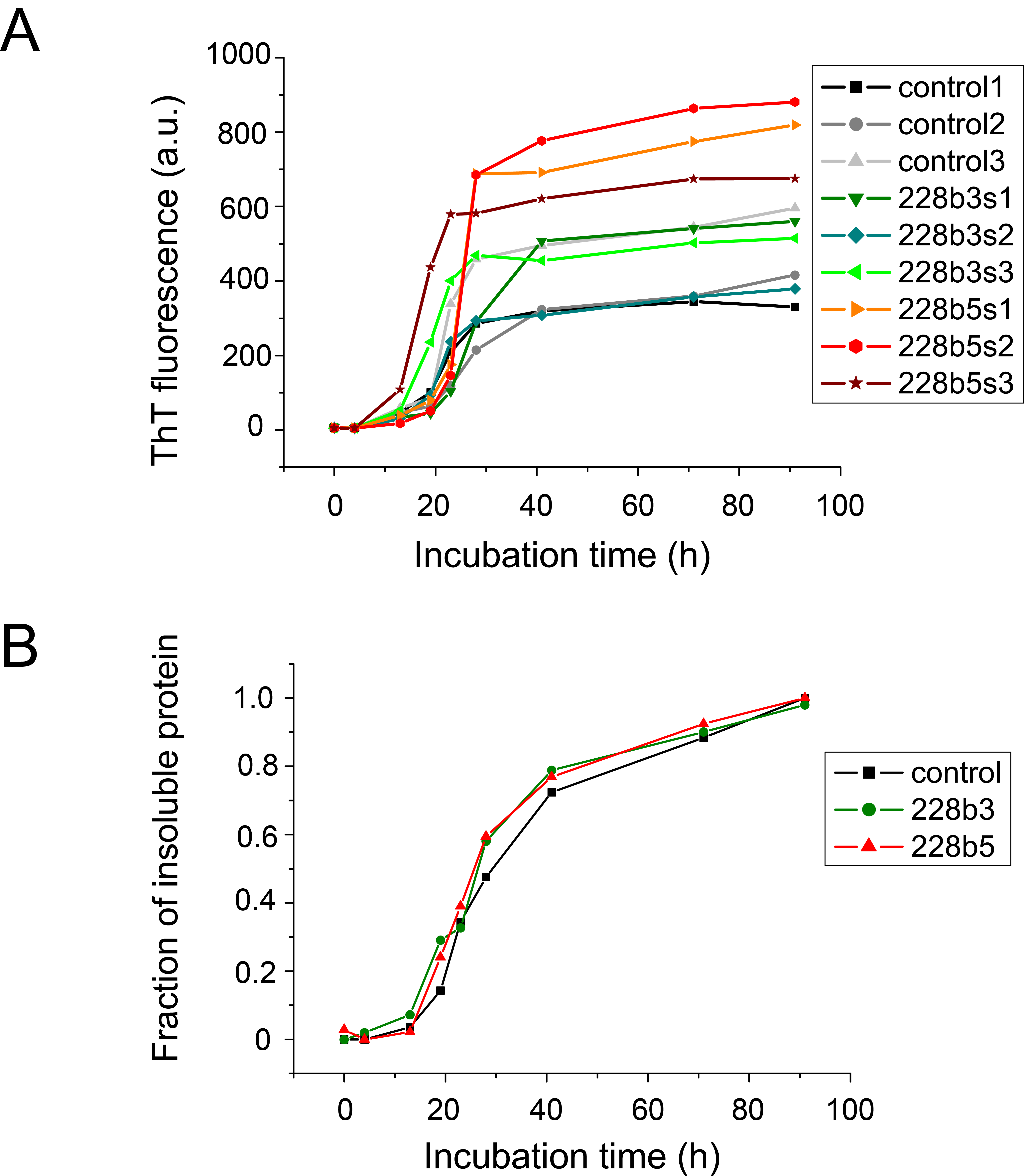

Supplement: Figure S3 — ELN484228 did not influence the aggregation kinetics of αSyn amyloid formation. Aggregation of αSyn in the presence of 484228 compared to the corresponding DMSO control following both (a) ThT signal and the fraction of soluble protein remaining in solution according to (b) SDS-PAGE electrophoresis. (Control1-3 is DMSO. 228b3as1-3 and 228b35s1-3 are two distinct batches of 484228). Fibrils of αSyn grown in the presence of 484228 retain the characteristic amyloid morphology (data not shown). 484228 also did not impact aggregation in a more quantitative assay in which fibril seeds were added at the initiation of the assay (data not shown). (TIF) [file pone.0087133.s004.tif]

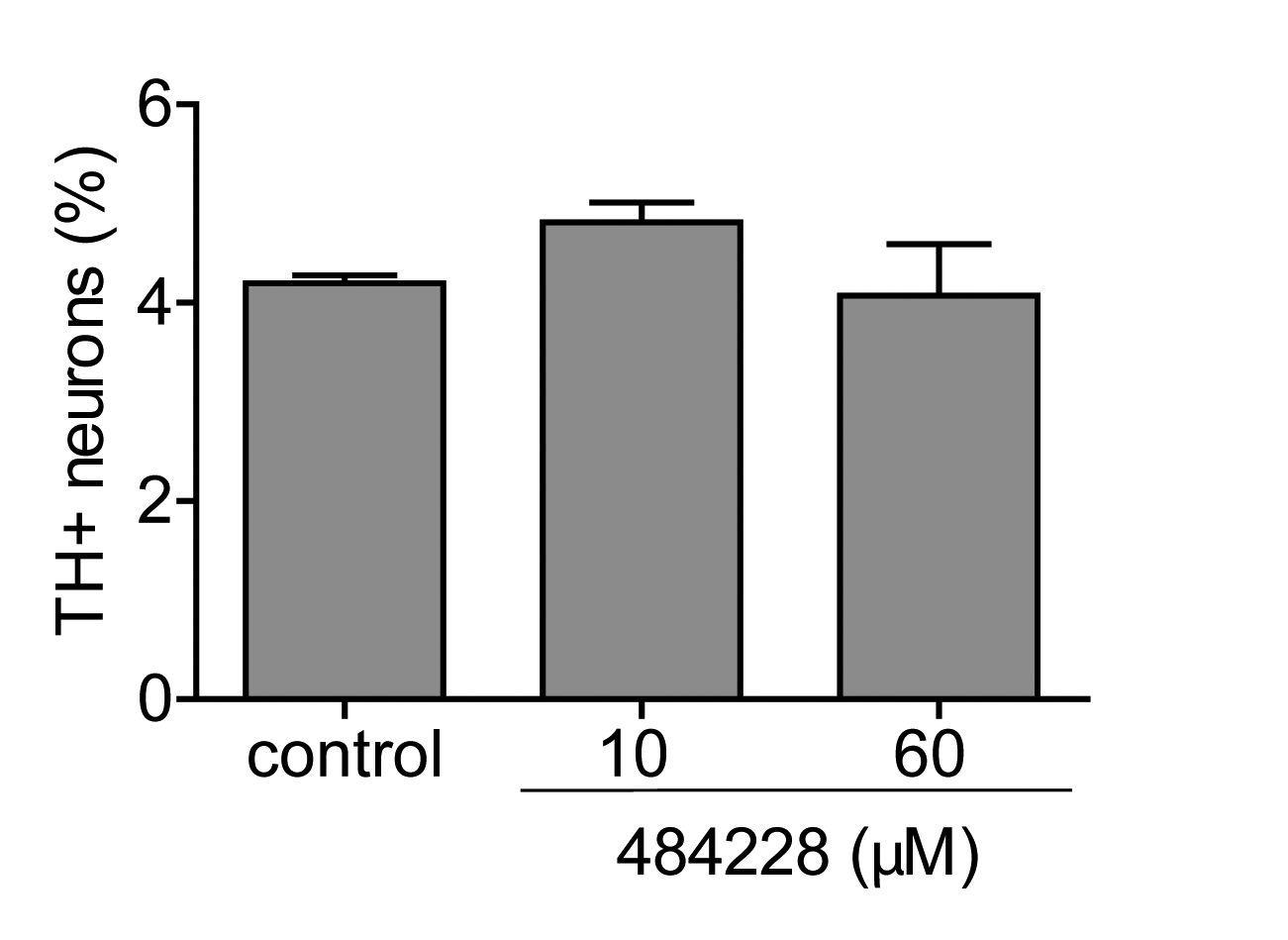

Supplement: Figure S4 — Compound 484228 is not cytotoxic at concentrations up to 60 µM. Primary midbrain cultures from E17 rat embryos were incubated with or without the compound for 96 h. The data are plotted as the mean ± s.e.m. (n = 2). (TIF) [file pone.0087133.s005.tif]

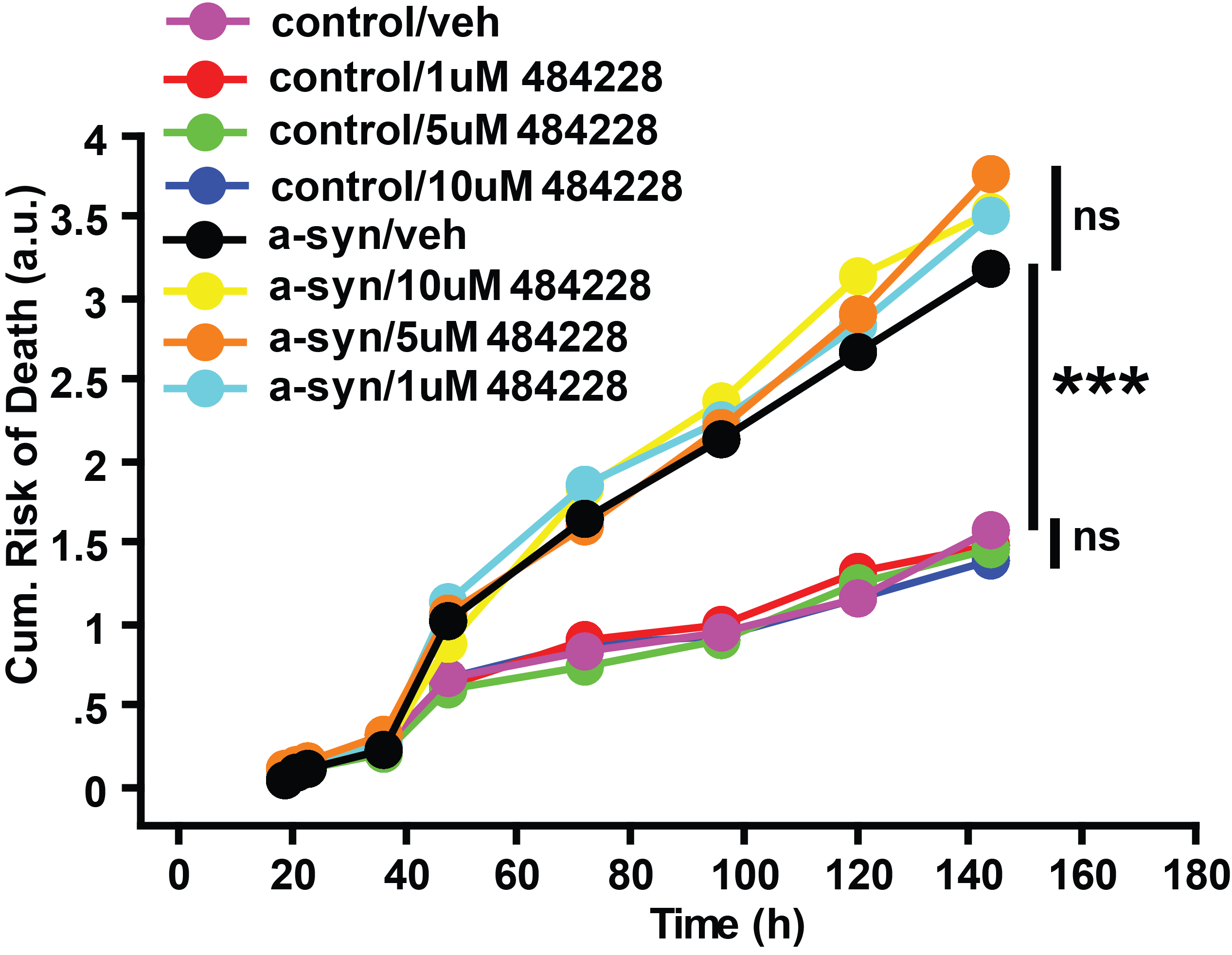

Supplement: Figure S5 — Increasing Concentrations of 484228 Does Not Reduce α-Syn mediated toxicity in cortical neurons. Cumulative risk of death curves demonstrate that cortical neurons overexpressing α-synuclein have a significantly increased risk of toxicity (α-syn/veh (veh = vehicle) versus control/veh, hazard ratio (HR) 1.61, p value < 0.0001). The exposure of cortical neurons overexpressing α-synuclein to increasing concentrations of ELN484228 did not significantly reduce α-synuclein mediated toxicity (α-syn/veh versus α-syn/1uM, HR 1.09, p = 0.31; α-syn/veh versus α-syn/5uM, HR 1.1, p = 0.31; α-syn/veh versus α-syn/10uM, HR 1.03, p = 0.79). Exposure of control cells to increasing concentrations of 484228 did not cause toxicity (control/veh versus control/1uM, HR 1.02, p = 0.87; control/veh versus control/5uM, HR 0.95, p = 0.59; control/veh versus control/10uM, HR 0.95, p = 0.63). Number of neurons α-syn/veh = 287, α-syn/1uM = 237, α-syn/5uM = 258, α-syn/10uM = 206, control/veh = 276, control/1uM = 293, control/5uM = 226, control/10uM = 232, 3 independent experiments combined. (TIF) [file pone.0087133.s006.tif]
