# Supplementary material for: Correction: Targeting the Intrinsically Disordered Structural Ensemble of α-Synuclein by Small Molecules as a Potential Therapeutic Strategy for Parkinson’s Disease
Source: PLoS One. 2014 May 27;9(5):e99274. doi: 10.1371/journal.pone.0099274 (PMC4035346; doi:10.1371/journal.pone.0099274)
Supplement: File S2 — Republished, corrected article. (PDF) [file pone.0099274.s002.pdf]

OPEN ACCESS Freely available online

PLOS ONE

# Targeting the Intrinsically Disordered Structural Ensemble of α-Synuclein by Small Molecules as a Potential Therapeutic Strategy for Parkinson's Disease

**Gergely Tóth[1,4]*, Shyra J. Gardai[4], Wagner Zago[4], Carlos W. Bertoncini[1,2], Nunilo Cremades[1], Susan L. Roy[3], Mitali A. Tambe[3], Jean-Christophe Rochet[3], Celine Galvagnion[1], Gaia Skibinski[6,7], Steven Finkbeiner[6,7,8], Michael Bova[4], Karin Regnstrom[4], San-San Chiou[4], Jennifer Johnston[4], Kari Callaway[4], John P. Anderson[4], Michael F. Jobling[4], Alexander K. Buell[1], Ted A. Yednock[4], Tuomas P. J. Knowles[1], Michele Vendruscolo[1]*, John Christodoulou[5], Christopher M. Dobson[1]*, Dale Schenk[4]*, Lisa McConlogue[4]***

**1** Department of Chemistry, University of Cambridge, Cambridge, United Kingdom, **2** SEDIPFAR (Servicio De Descubrimiento, Diseño Y Desarrollo Pre-Clínico De Fármacos De La Argentina) drug discovery platform, Universidad Nacional de Rosario, Rosario, Argentina, **3** Department of Medicinal Chemistry and Molecular Pharmacology, Purdue University, West Lafayette, Indiana, United States of America, **4** Elan Pharmaceuticals, South San Francisco, California, United States of America, **5** Department of Structural & Molecular Biology, University College London, London, United Kingdom, **6** Gladstone Institute of Neurological Disease, San Francisco, California, United States of America, **7** Taube-Koret Center for Neurodegenerative Disease Research, The Consortium for Frontotemporal Dementia Research, and The Hellman Family Foundation Program for Alzheimer's Disease Research, San Francisco, California, United States of America, **8** Departments of Neurology and Physiology, UCSF, San Francisco, California, United States of America

## Abstract

The misfolding of intrinsically disordered proteins such as α-synuclein, tau and the Aβ peptide has been associated with many highly debilitating neurodegenerative syndromes including Parkinson's and Alzheimer's diseases. Therapeutic targeting of the monomeric state of such intrinsically disordered proteins by small molecules has, however, been a major challenge because of their heterogeneous conformational properties. We show here that a combination of computational and experimental techniques has led to the identification of a drug-like phenyl-sulfonamide compound (ELN484228), that targets α-synuclein, a key protein in Parkinson's disease. We found that this compound has substantial biological activity in cellular models of α-synuclein-mediated dysfunction, including rescue of α-synuclein-induced disruption of vesicle trafficking and dopaminergic neuronal loss and neurite retraction most likely by reducing the amount of α-synuclein targeted to sites of vesicle mobilization such as the synapse in neurons or the site of bead engulfment in microglial cells. These results indicate that targeting α-synuclein by small molecules represents a promising approach to the development of therapeutic treatments of Parkinson's disease and related conditions.

**Citation:** Tóth G, Gardai SJ, Zago W, Bertoncini CW, Cremades N, et al. (2014) Targeting the Intrinsically Disordered Structural Ensemble of α-Synuclein by Small Molecules as a Potential Therapeutic Strategy for Parkinson's Disease. PLoS ONE 9(2): e87133. doi:10.1371/journal.pone.0087133

**Editor:** Mark R. Cookson, National Institutes of Health, United States of America

**Received** September 24, 2013; **Accepted** December 19, 2013; **Published** February 14, 2014

**Copyright:** © 2014 Tóth et al. This is an open-access article distributed under the terms of the Creative Commons Attribution License, which permits unrestricted use, distribution, and reproduction in any medium, provided the original author and source are credited.

**Funding:** Work at Elan and some of the work at the University of Cambridge was funded by Elan Pharmaceuticals. Additional funding came from the Wellcome Trust, EU-FP7 Marie Curie Fellowship, a Ramón y Cajal research contract from MICINN-Spain, a Human Frontier Science Program Long-term Fellowship and the NIH/NINDS. The funders had no role in study design, data collection and analysis, decision to publish, or preparation of the manuscript.

**Competing Interests:** We have read the journal's policy and have the following conflicts: some of the authors were employees and/or shareholders of Elan Pharmaceuticals as designated by Elan affiliation. Additional authors were supported in part by a grant from Elan Pharmaceuticals (CWB and NC). This does not alter our adherence to all the PLOS ONE policies on sharing data and materials.

* E-mail: lisam@msg.ucsf.edu (LM); gt293@cam.ac.uk (GT); mv245@cam.ac.uk (MV); dale.schenk@prothena.com (DS); cmd44@cam.ac.uk (CMD)

## Introduction

The aggregation and accumulation of α-synuclein (αSyn) constitutes the hallmark of a group of neurodegenerative disorders, often referred to as synucleinopathies, which include Parkinson's Disease (PD) [1]. In PD, αSyn accumulates in Lewy bodies as amyloid fibrils [2]. In addition, αSyn gene multiplications or missense mutations cause rare early onset forms of PD [3,4,5,6,7] and genetic association studies link αSyn to sporadic PD [8,9]. Together, these findings strongly implicate αSyn as a key factor in the pathophysiology of PD. Monomeric αSyn is an example of an intrinsically disordered protein (IDP). IDPs can be represented as ensembles of interconverting conformations and are involved in many key biochemical processes [10,11], as well as in a growing number of misfolding diseases, notably the most common forms of neurodegenerative conditions [12,13].

A promising approach to remedying the aggregation and functional perturbations consequent to protein misfolding in disease is to use small molecule binding to stabilize the native states of proteins [14]. The development of such compounds has been successful in the case of globular proteins, such as transthyretin [15,16], a protein whose misfolding leads to systemic amyloidosis and related disorders, and glucocerebrosidase, a protein for which misfolding leads to Gaucher disease [17]. The structural heterogeneity and lack of persistent structural elements

for αSyn and other IDPs, however, pose a major challenge in the discovery and design of small molecules for these proteins [18,19].

*In silico* screening methods based on high-throughput docking of large small molecule libraries to natively folded proteins have become an increasingly important strategy for initial lead discovery as a consequence of the wide availability of high-resolution structures of protein targets and of the rapid advancement of computational and screening methods [19,20]. Applying such methods to IDPs, such as αSyn is, however, not straightforward because of the lack of a well-defined target structure. Nevertheless, despite the absence of persistent structural elements, the conformational space populated by αSyn is not random and can be characterized at relatively high resolution by combining NMR spectroscopy with advanced structure computational methodologies [10,21,22]. These studies have revealed that αSyn populates a wide range of conformations in solution, including relatively compact but transient states resulting from the presence of residual local structure and long-range contacts within the fluctuating structural ensemble [23,24]. Such residual local structure could provide an opportunity for small molecule discovery if appropriate binding pockets exist.

Such an expectation is based on the premise that small molecule binders to αSyn have the potential to bind to specific conformations present in the ensemble and modify its properties, which in turn could influence the way in which αSyn molecules interact among themselves and with other proteins. Thus, depending on which conformations of αSyn are involved, binding molecules could alter its aggregation propensity, or perturb the malfunction or the toxicity of αSyn that are associated with its over-expression [25,26,27,28,29,30,31,32,33,34,35,36]. Therefore, we set out to explore the potential of a drug discovery strategy designed to identify small molecules that can bind αSyn and then to test them in a diverse set of PD-relevant *in vitro* biochemical and cellular assays to look for potentially beneficial effects.

In addition to aggregation and toxicity resulting from αSyn misfolding, elevation of αSyn levels is also associated with the dysfunctional formation, localization, and maintenance of cellular vesicle pools [26,31,32,34,36]. A role for αSyn has been established in vesicle dynamics including impact on synaptic vesicle [27,28,29,33,34], ER to Golgi trafficking [30] and exocytosis in both neuronal and non-neuronal cells [26,32,36]. Under physiological conditions, αSyn enhances neurotransmission through its effects on synaptic vesicle exocytosis or endocytosis [25]. In contrast, overexpression of αSyn affects select synaptic vesicle pools and reduces neurotransmission [31,34], and there is evidence for a link between the impact of αSyn on vesicle dynamics and αSyn-mediated toxicity [26]. The ability of αSyn to modulate membrane curvature, and the impact of αSyn on SNARE function have been postulated to be the underlying molecular mechanism by which αSyn plays such diverse roles in vesicle dynamics [37,38,39]. We have recently shown that phagocytosis, a process dependent on membrane expansion via focal vesicle extrusion to engulf particles [40,41], is impaired in cells and mice over-expressing αSyn and also in fibroblasts and blood cells from PD patients [42]. Thus, impairment of phagocytosis by elevated αSyn provides a cellular model of αSyn malfunction associated with its role on vesicle dynamics and in PD [42].

In the present work we predicted small molecule binding sites present in a subset of αSyn conformations populated by the monomeric protein and screened for small molecules binding to these sites using an *in silico* structure-based computational docking screen. The application of this approach resulted in the discovery of several small-molecules predicted to bind to αSyn, among which, one compound (ELN484228), was characterized in particular detail in biochemical and cellular models. We have found that ELN484228 reverses αSyn-induced impairment of phagocytosis and protects dopaminergic neurons against the toxic effects of αSyn A53T over-expression. As a control we show that another compound, ELN484217, which is predicted to bind to a very different conformation of αSyn, does not impact αSyn-induced impairment of phagocytosis. We thus provide evidence supporting a potential route for the development of therapeutic strategies for PD; in which αSyn is targeted by small molecules that can reverse αSyn-mediated malfunction.

## Materials and Methods

A full description of methods can be found in the Supporting Information text file S1.

### Ethics Statement

All animal work was conducted according to relevant national and international guidelines under protocols approved by Elan's Institutional Animal Care and Use Committee.

### Molecular Modeling

Energy minimization of αSyn and small-molecule conformations and identification of hot spots are described in the Supporting Information text file S1 (Table S1 and Table S2). Docking calculations for a small-molecule library of 33,000 fragment-like compounds were carried out using FRED 2.0 (OpenEye Scientific Software, Inc., Santa Fe, www.eyesopen.com, 2008 and [43], See a full description of docking method in the Supporting Information text file S1). After docking calculations, AM1/BCC charges were calculated for each ligand conformation and used in the subsequent minimization step. Optimization of the ligand in the binding site was performed by minimization of each docked conformation using SZYBKI 1.2.0 (OpenEye Scientific Software, Inc., www.eyesopen.com, 2008). During the SZYBKI minimization the MMFF94 molecular mechanics force field and the Poisson-Boltzmann solvation model was used. The protein structure was kept rigid while all the atoms of the ligand were allowed to be flexible.

Identification of hot spots and binding pockets was done using a computational fragment probe mapping methodology [19,44,45] (see also the Supporting Information text file S1). During this process, 15 different small molecules (fragment probes) (Fig. 1B) were docked onto the surfaces of the whole conformational set as described in the Supporting Information text file S1. The set of fragment probes used here contained diverse functional groups and shapes, which enabled them to bind to a variety of hot spots and binding sites. Hot spots were ranked using the potential ligand efficiency ($L_e$) score [19]. $L_e = \sum[E_{vdW}/$(number of heavy atoms in a fragment probe)]/(number of fragment probes that bind the hotspot). Hot spots were located where the strongest interacting different fragment probes clustered. A potential binding pocket was identified when two or more high-ranking hot spots were in close proximity of each other. In the following we analyze two compounds (ELN484228 and ELN484217), which were identified through this procedure and are predicted to bind to two very different conformations of αSyn (Fig. S1).

### αSyn Purification

Recombinant αSyn was produced in *E. coli* and purified under heat-denatured conditions using standard protocols [46,47,48].

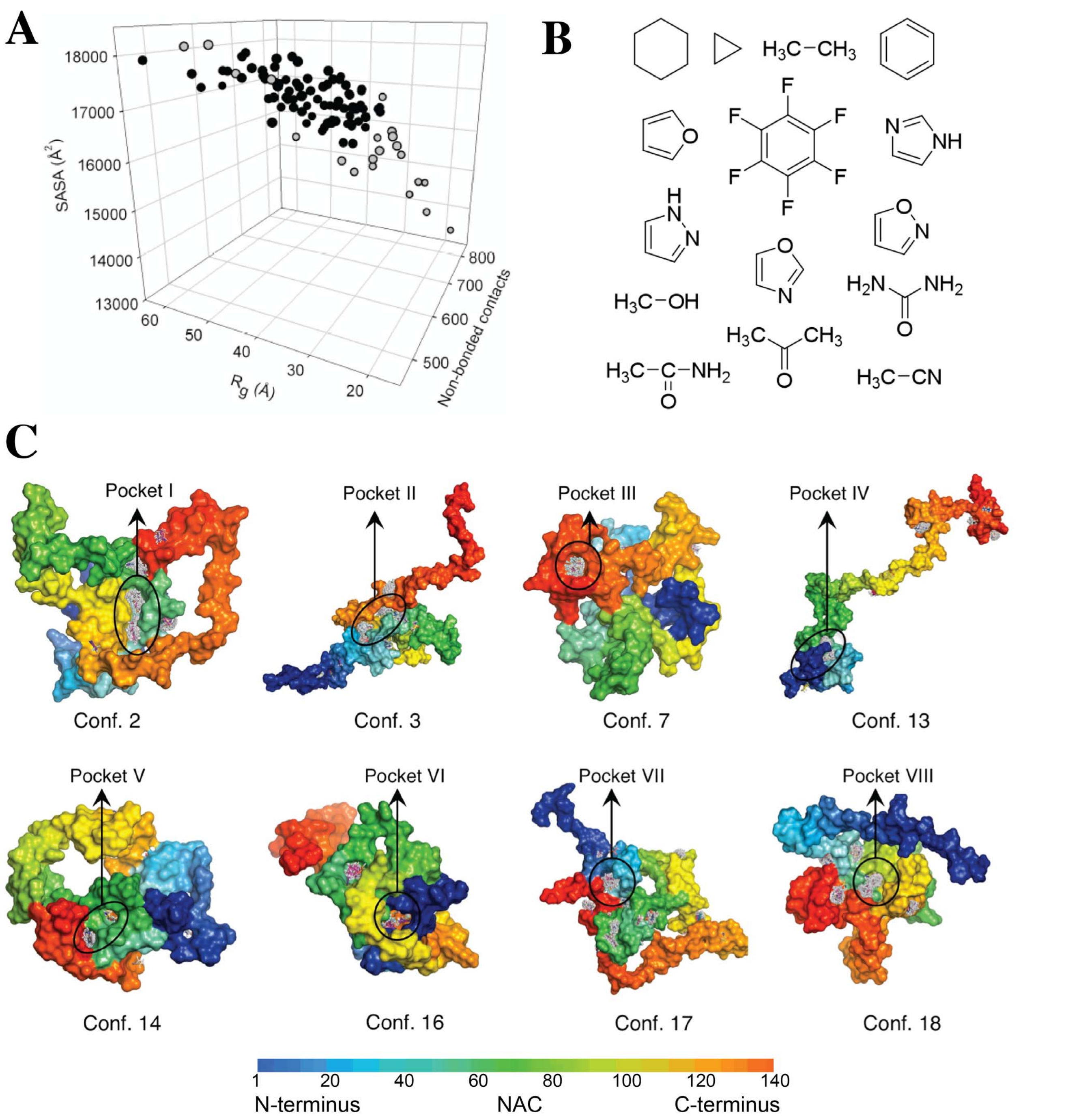


**Figure 1. Fragment probe mapping identifies potential small-molecule binding pockets in monomeric αSyn. A.** Structural properties of the 100 analyzed conformations from the NMR-derived ensemble structure of αSyn; correlation between the radius of gyration (Rg), solvent accessible surface area (SASA) and the number of non-bonded contacts. The gray circles represent the αSyn conformations, which were used for the fragment probe mapping calculations. **B.** 2D chemical structure of small molecules used as fragment probes in the fragment probe mapping calculations. **C.** 3D representation of the eight pockets identified within αSyn conformations filled with clusters of small molecules resulting from the fragment probe mapping calculations. Residues of αSyn are colored according to the amino acid sequence.
doi:10.1371/journal.pone.0087133.g001

## Aggregation Assays

Aggregation of αSyn was assayed in triplicates at 37°C under shaking (300 rpm) in solutions containing 50 μM protein in the absence and presence of tenfold higher concentration of compound ELN484228 in 25 mM Tris buffer pH 7.4, 100 mM NaCl with the addition of 0.01% $NaN_3$. Aliquots were withdrawn on a daily basis and the thioflavin T (ThT) fluorescence signal was recorded after addition of 20 μM of ThT to each aliquot. Fluorescence emission spectra from 460 to 600 nm were then recorded at an excitation wavelength of 446 nm employing a Cary-Eclipse spectrofluorimeter (Varian, Palo Alto CA). Quenching of the ThT fluorescence by the addition of ELN484228 was

assayed by incubating pre-formed fibrils with the compound and by comparison of the ThT fluorescence signal before and after the addition of ELN484228, but no significant change in signal was found. The aggregation of αSyn in the presence of ELN484228 was also characterized in the presence of low concentrations of SDS (200 μM) under the same experimental conditions as described above. The time-dependences of the ThT fluorescence signal were fitted to a nucleation-elongation model as previously described [49]. TEM images were obtained using a Philips CEM100 transmission electron microscope. The samples were applied on Formvar-carbon coated nickel grids and stained with 2% (w/v) uranyl acetate.

## Transgenic Animals

A full description of the generation of transgenic animals is provided in the Supporting Information text file S1 and [42]. Briefly, Bacterial Artificial Chromosome (BAC) clone RP11-458H10, containing the human SNCA gene sequence (Life Technologies, Carlsbad, CA) was modified to generate both the Rep1 mutation [50] and E46K mutation [51] by BAC recombineering methods as described [52]. Circular BAC constructs containing the hSNCA transgene (~168 Kb) were used to perform pronuclear microinjection into B6SJL F2 mouse strains (The Jackson Laboratories, Bar Harbor, ME) in the concentration of 1–3 μg/μl followed by implantation into pseudo pregnant females (Xenogen Biosciences, Cranbury, NJ). Founder animals were bred with B6D2F1 mice and maintained as heterozygotes on this background with non-transgenic littermates as controls. Line BAC-Tg3(SNCA$^{E46K}$) animals were bred in sufficient numbers for a 3, 8–9, 12–14, 18–20 month old (MO) characterization cohorts and were 3–8 generations from founders. All mice were housed in a pathogen-free, climate controlled and given food and water ad libitum. All animal studies were reviewed and approved by the Institutional Animal Care and Use Committee at Elan pharmaceuticals in accordance with the National Institutes of Health Guide for the Care and Use of Laboratory Animals.

## Primary Cell Cultures

Microglia were obtained from cerebral cortices of 1–3 day old neonate mice. A full description of microglia culture methods is provided in the Supporting Information text file S1. Hippocampal neurons were isolated from embryonic day 18 prenatal rat hippocampi and cultured in antibiotic- and serum-free NbActiv4 medium (both from BrainBits, Springfield IL) at 37°C in an atmosphere of 5% CO2, 9% O2 and on glass coverslips coated with poly-lysine. Half of the medium was replaced every 3 to 4 days. Cells were used for the experiments after 21–28 days *in vitro*.

Primary midbrain cultures were prepared from embryonic day 17 embryos of Sprague-Dawley rats using methods reviewed and approved by the Purdue Animal Care and Use Committee as described previously [30,35,53,54]. After dissection, the cells were plated into a poly-L-lysine-treated 48-well plate at a density of 163,500 cells per well. Four days later, the cells were treated with cytosine arabinofuranoside (AraC) (20 μM) for 48 hours to inhibit the growth of glial cells. The cultures were used for measurements of dopaminergic neuron viability and neurite length at 7 days *in vitro*.

Primary cortical neurons were prepared from embryonic rats. Timed pregnant rats were obtained from Charles River Laboratories (Cambridge, MA). Primary cultures of rat cortical neurons were prepared from rat pup cortices on embryonic days 20–21. Cortices were dissected and treated with papain (10 units/ml, 15 minutes; (Worthington)) followed by addition of trypsin inhibitor (10 mg/ml, 15 minutes; Sigma). After trituration, dissociated neurons were plated on plastic 24 well plates coated with laminin (BD Biosciences) and poly-D-lysine (Millipore). Neurons were grown in Neurobasal-A with B27 medium (Invitrogen) and were used for experiments at 5–7 days *in vitro* (DIV). Neurons were transfected with plasmids using lipofectamine2000. Neurons were co-transfected with pCAGGS control and pGW1-RFP or pCAGGS αSyn and pGW1-RFP, in a 1:8 molar ratio. ELN484228 was added to the neurons at 1, 5 and 10 μM concentrations. The compound was added to the neurons directly after transfection and again at 72 and 120 hours post transfection. DMSO was used as a vehicle control.

## Phagocytosis Assay

Isolated hSNCA$^{E46K}$ non-transgenic and αSyn transgenic mouse microglia were plated in 24-well plates at $1\times10^5$ cells/well for 1 day. Cells were then treated with various concentration of ELN484228 or ELN484217, another compound selected from the *in silico* hits (compound number 38 in table S4 in the Supporting Information text file S1), for 24 hours. 10 μm FluorSpheres particles, 5×10^6 10 μ beads (Life Technologies) were washed with 1 ml of PBS 3 times to remove azide and added (1:10) for 90 minutes at 37°C. Unbound particles were washed away with PBS and cells were stained with Wright's Giemsa (Leukostat; Fisher, Freemont CA) for 30 seconds for each stain and phagocytosis was determined by light microscopy. A phagocytic index was calculated using the formula PI = (# particles ingested/#cells counted)×100. 200 cells were visualized for each well and each condition was performed blinded in duplicate. A human neuroglioma H4 cell-derived cell line stably over-expressing αSyn from a tetracycline inducible promoter was generated using the tet inducible TRex system (Invitrogen division of Life Technologies) and selecting the clone with maximal induction and expression. For assessment of phagocytosis in H4 cells, cells were plated at 25,000 cells per 24 well plate and following an overnight incubation were treated with various concentration of ELN484228 or ELN484217 and tetracycline at 1 μg/ml to induce αSyn overexpression and cultured for an additional 24 hours. 4 μm FluorSpheres particles, (Life Technologies) were added at a ratio of 1:10 for 90 minutes, to assess phagocytotic capacity. Cells were fixed, stained and phagocytosis assessed as above.

## Microscopy Analysis

For αSyn translocation analysis, H4 cells were plated and treated as in the phagocytosis assay, and the cells were fed 4 μm beads for 90 minutes. The cells were fixed with 4% PFA for 20 minutes, and then permeabilized with 0.5% saponin (Sigma-Aldrich, St. Louis, MO) and blocked with 1% BSA for 30 minutes on ice. 1 μg of primary antibody 5C12 was added overnight at 4°C, and cells were washed twice and incubated with 594-conjugated secondary antibody (1:100 dilution) for 30 minutes. Cell were washed twice in PBS and then stained for actin with 1:100 of Alexa488-phalloidin and the nuclear stain DAPI at 1:1000 for 30 minutes. Cells were visualized on an Olympus1X81 scope (Olympus, Center Valley NJ) with a 20X/0.45 objective or a 63X/0.70 objective. Digital images were taken using MetaMorph imaging software (Molecular Devices, Sunnyvale, CA).

For analysis of synaptic αSyn levels, primary hippocampal cultures were treated with 1 μM ELN484228 or the vehicle control (0.01% DMSO) in NbActiv4 media at 37°C for 24 hours. The cells were then fixed in 4% paraformaldehyde, permeabilized with 0.1% Triton X-100, and further incubated with primary antibodies for 24 hours at room temperature. These included a mouse anti-αSyn (5C12, Elan Pharmaceuticals [55]) and a rabbit

anti-synaptophysin (Millipore Corporation, Billerica, MA). The detection was performed after incubation with appropriate secondary antibodies conjugated to Alexa fluorophores (Life Technologies, InVitrogen). Any non-specific signal was determined by substituting primary antibodies with appropriate IgG controls. Digital images of fluorescently labeled cells were collected on a Leica SPE laser scanning confocal microscope (Leica microsystems, Wetzlar Germany) using a 63X objective. A total of 10 to 20 optical fields per group per experiment were randomly sampled by software. Fields containing fewer than 2 neurons were discarded. Images were analyzed with MetaMorph imaging system (Molecular Devices, Sunnyvale CA) and synaptic αSyn staining was determined by measuring the amount of fluorescent signal (average intensity) at the presynaptic sites, identified by the presence of the presynaptic marker synaptophysin.

### Robotic Microscope Imaging System, Image Analysis and Related Statistics

For imaging of primary cortical neurons to assess αSyn impact on survival, images of neurons were taken at 24-hour intervals after transfection using the automated microscope system previously developed [56]. Image acquisition and analysis were carried out on ImagePro Plus 6.2. Measurements of αSyn neuron survival were obtained from files generated with automated imaging and visual inspection. Living transfected neurons were selected for analysis based on fluorescence intensity and morphology. Neurons were only selected if they have extended processes at the start of the experiment. The survival time of a neuron was measured as the last time point at which the neuron was seen alive.

For statistical analysis of primary cortical neurons to assess αSyn impact on survival, Kaplan-Meier curves were used to estimate survival using statview software package. Hazard ratios and their respective p-values were generated using the coxph function in the survival package for R statistical software [57].

### Dopaminergic Neuron Viability Assay & Neurite Length Analysis

Primary midbrain cultures were transduced with αSyn A53T adenovirus (MOI = 10), in the absence or presence of ELN484228, as described previously [35]. After 72 hours, the cells were incubated with fresh media with or without the compound for another 24 hours prior to immunocytochemical analysis, which was carried out as described previously [30,35,53,54]. The cells were fixed, permeabilized, and blocked prior to an overnight treatment with two primary antibodies: a mouse monoclonal IgG specific for microtubule-associated protein 2 (MAP2) (1:500) and a rabbit polyclonal antibody specific for tyrosine hydroxylase (TH) (1:500). Next, the cells were treated with two secondary antibodies, goat anti-mouse IgG conjugated to AlexaFluor 594 and goat anti-rabbit IgG conjugated to AlexaFluor 488 (1:1000) for 1 hour. In order to determine the viability of dopaminergic neurons, MAP2- and TH-positive neurons were counted in 10 to 15 randomly chosen observation fields in a blinded manner using a Nikon TE2000-U inverted fluorescence microscope (Nikon Instruments, Melville NY) with a 20X objective. In the control conditions, and in conditions where the compound was neuroprotective, we typically counted 500 to 1,300 MAP2-positive neurons, a range that corresponds to 20 to 50 TH–positive neurons [53]. The data were expressed as the ratio of the TH-positive neurons to the MAP2-positive neurons. Each experiment was repeated at least three times using embryonic midbrain cultures from different pregnant rats. The neurite length analysis was carried out on the same samples used for the dopaminergic neuron viability assay. Overlay images of MAP2- and TH-positive neurons were taken in a blinded manner using a Nikon A1 confocal microscope (Nikon Instruments, Melville NY), and neurite lengths were measured for 160 to 206 neurites per treatment (corresponding to ≥78 neurons pooled from three independent experiments) using the NIS-Elements software. For both the neuron viability assay and the neurite length analysis, statistical analyses were carried out using the program GraphPad Prism, Version 6.0 (http://www.graphpad.com/prism/Prism.htm).

## Results

### Computational Identification of small Molecule Binders of αSyn

Our strategy to identify small molecule binders of αSyn is based on the description of the structural properties of IDPs in terms of conformational ensembles [19,23,24,58,59]. Here, we used an ensemble of structures determined by a combination of NMR and computational methods [24] as the basis for a three-step *de novo* computational screen to identify potential small molecule binders to αSyn. In the first step, 100 structures were randomly chosen from an ensemble of 40,000 NMR-derived αSyn conformations (Fig. 1A). From this set, 22 diverse members were identified by a selection process with some bias towards the more compact conformations (open circles in Fig. 1A; fully described in the Supporting Information text file S1 and Fig. S1), as these are more likely to offer binding pockets that are more suited for the binding of small molecules. In the second step, computational fragment probe mapping [44] was used to identify "drugable hot spots" located where the strongest interacting different fragment probes clustered (see Materials and methods and Supporting Information text file S1) [60], on these 22 αSyn conformations (Fig. 1B). This procedure led to the identification of eight binding pockets, each of which is located in a different member of the αSyn ensemble (Fig. 1C and Table S2). Most of the sites identified are present at an interface involving relatively long-range tertiary contacts (Table S3) between different regions of αSyn. In the third step, a library of drug-like small molecules, containing approximately 33,000 commercially available fragment-like compounds having a molecular weight under 325 Da, were docked individually to each of the eight different binding pockets. These calculations resulted in the identification of 89 compounds (referred to as *in silico* hits), representing a small fraction of the screened molecules as potential ligands for these binding pockets (between 9 to 18 binders for each pocket, table S2 and Fig. S2). These compounds were considered suitable candidates for an experimental test of their ability to impact αSyn (Table S4). We chose one compound, ELN484228 (entry number 7 in Table S4), for further study in αSyn aggregation assays and in cellular models of αSyn malfunction, and as a control we chose another compound, ELN484217 (entry number 38 in Table S4), which is predicted to bind to a very different conformation of αSyn than that of ELN484228 as ELN484228 is predicted to bind to pocket I and ELN484217 to pocket IV (Table S4 and Fig. 1).

### ELN484228 is Protective in Cellular Models of αSyn-mediated Vesicular Dysfunction

Although ELN484228 did not detectably modify αSyn aggregation *in vitro* when probed by ThT fluorescence (Fig. S3), the screening procedure that we used has the potential to identify compounds affecting other αSyn mediated dysfunctions. αSyn overexpression can alter vesicular dynamics in cells including the impairment of phagocytosis [42]. We tested the effects of the compound in two cellular models in which αSyn overexpression

results in defective phagocytosis. Both H4 neuroglioma cells overexpressing wildtype αSyn in a tetracycline regulatable manner (H4-syn) and microglia isolated from αSyn BAC transgenic animals overexpressing the E46K mutant form of human αSyn display impaired phagocytosis [42]. Previous experiments demonstrated that overexpression of control proteins including β-synuclein has no impact on phagocytosis [42].

H4-syn cells were exposed to ELN484228 or another compound selected from the *in silico* hits, ELN484217, followed by tetracycline-induced overexpression of αSyn. Cells were fed 4 μm beads for 90 minutes and a phagocytic index was measured. Multiple Western blot measurements demonstrate that levels of αSyn were unchanged by the addition of ELN484228 (data not shown). However, treatment with ELN484228, but not ELN4848217, restored phagocytosis in the αSyn overexpressing cells in a dose dependent manner, with a 30 μM solution restoring phagocytosis by 60%, and complete rescue of the αSyn mediated phagocytosis deficit occurring with 100 μM ELN484228 (Fig. 2A). ELN484228 did not alter phagocytosis in cells not overexpressing αSyn. Restoration of phagocytosis following ELN484228 treatment was also assessed in αSyn overexpressing microglia isolated from αSyn BAC transgenic animals. Similar to what was observed in the H4-syn cells, ELN484228, but not ELN4848217, treatment restored phagocytosis to that observed in wild type microglia (Fig. 2B) without altering overall αSyn levels (data not shown). Thus, in two different cellular paradigms, ELN484228 reverses the impairment of phagocytosis mediated by αSyn whereas ELN484217, which is predicted to bind to a different conformation of αSyn, has no impact on phagocytosis.

## ELN484228 Alters Targeting of αSyn to the Phagocytic Cup and to Synapses

Previously we demonstrated that under resting conditions αSyn is evenly distributed in the cytoplasm of H4-syn cells, and that following the addition of beads, αSyn is recruited to the phagocytic cup [42]. The inhibition of phagocytosis by overexpressed αSyn is accompanied by altered vesicle mobilization, SNARE complex assembly and membrane extrusion [42], which may be a result of the elevated αSyn levels at the phagocytic cup. The consequence of ELN484228 treatment on αSyn recruitment to the phagocytic cup was assessed in αSyn overexpressing cells after 24 hours of compound treatment. Fig. 2D shows that whereas αSyn was recruited to the site of bead binding and ingestion in untreated H4 cells overexpressing αSyn, treatment with ELN484228 prevents the redistribution of αSyn to the phagocytic cup, looking much more like the distribution observed in non-bead treated αSyn overexpressing H4 cells. Thus the accumulation of αSyn at the phagocytic cup under overexpressed conditions is prevented by ELN484228 and may contribute to the mechanism of protection in this model.

Reminiscent of the recruitment of αSyn to the phagocytic cup is the demonstrated recruitment of αSyn to the presynaptic terminal of mature synapses, where it regulates distinct pools of synaptic vesicles and when over-expressed impairs neurotransmitter release [28,33,34]. To extend our observations to neuronal systems, we first tested the impact of ELN484228 on the targeting of αSyn to synapses. Mature hippocampal neuronal cultures were treated with 1 μM of the compound and the synaptic levels of the protein measured by immunofluorescence. The assessment of the synaptic levels of αSyn was performed using an imaging-based procedure that measures the co-registration of the signal for the αSyn protein with that of the pan-presynaptic marker synaptophysin. Fig. 2E demonstrates that ELN484228 reduces αSyn targeting in rat primary neuronal cultures by 50%. A similar two-fold reduction in αSyn synaptic targeting was also seen in cortical cultures from human αSyn overexpressing transgenic mice and at doses of ELN484228 over a ten-fold dose range (data not shown). There were no changes in number of puncta or in mean intensity of synaptophysin/punctum (data not shown), indicating that the effect of the compound was directed to αSyn and did not involve synaptic remodeling. Western blot analyses demonstrate that overall levels of αSyn were unchanged by ELN484228 treatment (data not shown). This concentration of ELN484228 did not cause evident toxicity as measured by counting neuronal profiles and by monitoring synaptic density (synaptophysin). Signs of toxicity were observed at compound concentrations above 30 μM, where the loss of neurons was evident in light microscopy. As these experiments demonstrated impact at 1 μM compound in the absence of serum, we also tested restoration of phagocytosis in αSyn overexpressing H4 cells and showed that in the absence of serum ELN484228 also shows similar efficacy at 1 μM (data not shown). Thus we show that ELN484228 reduces the recruitment of αSyn to both the phagocytic cup and to mature synapses.

## ELN484228 is Protective against αSyn-mediated Degeneration in Dopaminergic Neurons

To test the effects of ELN484228 on αSyn-induced neurotoxicity, we used a primary midbrain culture model of PD [54]. As a control, we first established that there is no detectable toxic effect on primary dopaminergic neurons exposed to 10 μM ELN484228 alone (Fig. S4). Transduction of the primary cultures with an adenovirus encoding A53T αSyn, a PD-linked genetic mutant found to form toxic oligomeric species more readily than the wild type protein [5,61], results in a 30–40% reduction in the number of TH-immunoreactive neurons and a significant reduction in the length of neurites staining positive for both TH and MAP2 (Fig. 2C). In contrast, transduction with a virus encoding the control protein LacZ has no effect on neuron viability or neurite length [53] (data not shown). In the presence of 10 μM ELN484228, however, the neurotoxicity induced by A53T αSyn was found to be significantly suppressed, and TH-positive neuron viability was restored almost to the levels observed in non-transduced or vector control transduced cultures (Fig. 2C). In addition, treatment with 10 μM ELN484228 rescued the neurite retraction elicited by A53T αSyn in TH- and MAP2-positive neurons (Fig. 2C). The magnitude of the neuronal rescue by ELN484228 is similar to that exhibited by Rab GTPases [30] and SIRT2 inhibitors [62] when examined in this assay showing that ELN484228 has substantial neuroprotective activity. We did not, however, observe protection in a distinct model of αSyn mediated neuronal death in which immature rat cortical primary neurons transiently transfected to overexpress αSyn are individually followed and scored for viability using a robotic microscope [63,64] (Fig. S5). This result could be a consequence of the lack of synapses in this alternative model at this early stage of primary culture as neuronal death is seen early within the 5 day imaging protocol [63,64]. The mechanism of αSyn neurotoxicity is likely to be different in this model compared to that in the primary midbrain culture model, and is apparently not targeted by the compound.

# Discussion

We have pursued a novel discovery strategy to identify drug-like small molecules capable of binding monomeric αSyn and then analyzed the effects of select small molecules resulting from this procedure in a diverse set of PD relevant biochemical and cellular assays. An advantage of this screening strategy is its potential to

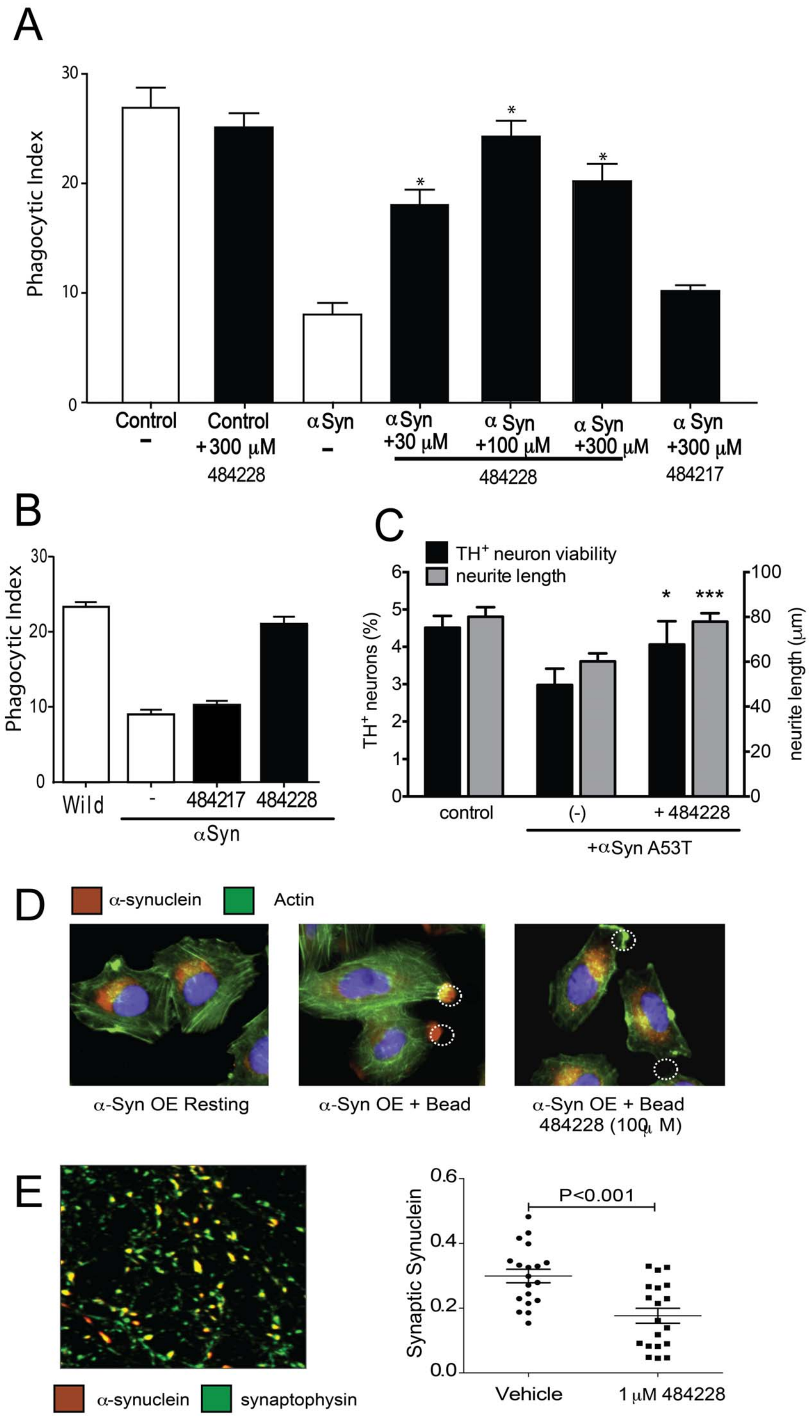


**Figure 2. ELN484228 provides protection in cellular models of αSyn-mediated dysfunction. A.** ELN484228 alleviates αSyn-mediated impairment of vesicular dynamics. H4 neuroglioma cells over-expressing αSyn from a tetracycline inducible promoter were cultured for 24 hours in the absence or presence of 1 μg/ml tetracycline to induce αSyn and ELN484228 or control compound ELN484217 (compound number 38 in table S4 in Supporting Information text file S1). Open bars: without compound, black bars: with indicated amount of compound. Cells were assayed for phagocytic activity as a measure of αSyn-mediated impairment of vesicular function. 4 μ beads were added for 90 minutes and a phagocytic index was calculated by microscopic visualization. Each sample was run in triplicate and experiments were run three independent times. The phagocytic indices for each individual experiment were averaged and statistical analyses run on the final averages from the three experiments. T-test analysis of the combined averages of the three experiments revealed a significant difference in phagocytosis between Tet-induced samples with and without ELN484228 (n = 3+/− s.e.m *p≤0.001 versus no compound Tet-induced sample). **B.** Microglia isolated from postnatal day 1 to 3 pups from hSNCA$^{E46K}$ transgenic (αSyn ) or non-transgenic littermates were incubated for 24 hours with 100 μM ELN484217 or ELN484228 followed by addition of 10 μm beads for 90 minutes. A phagocytic index was calculated by microscopic visualization (n = 3+/− s.e.m *p≤0.001). **C.** ELN484228 alleviates loss of dopaminergic neurons and neurite retraction induced by the A53T mutant of αSyn. Primary rat embryonic midbrain cultures were non-transduced ('control') or transduced with adenovirus encoding A53T αSyn, in the absence or presence of 10 μM ELN484228. The cells were then stained immunocytochemically for MAP2 and TH. Preferential dopaminergic cell death was assessed by evaluating the percentage of MAP2-positive cells that

also stained positive for TH. The lengths of neurites staining positive for both MAP2 and TH were measured using the NIS-Elements software. Data are plotted as the mean ± s.e.m. n = 3 for neuron viability analysis; n = 160–206 for neurite length analysis. *p-value≤0.05, ***p-value≤0.001 versus aSyn A53T virus in the absence of compound; one-way ANOVA with Newman-Keuls post-test. **D.** ELN484228 reduces translocation of αSyn to the phagocytic cup. To assess αSyn translocation, H4 cells were treated with 100 μM ELN484228 and 1 μg/ml tetracycline for 24 hours; cells were then stimulated with 4 μ beads for 90 minutes. Samples were fixed and stained with 5C12 antibody to detect αSyn (red). Cells were counterstained with 488-phalloidin (green) and Hoechts (blue). A dotted circle indicates the position of the bead. **E.** ELN484228 reduces translocation of αSyn to synapses. Rat hippocampal neurons (~21DIV) grown in serum-free conditions were treated for 24 hours with 1 μM ELN484228 or 0.01% DMSO vehicle. On the left side is a representative confocal microscopic image showing localization of αSyn (red) detected with 5C12 antibody, and localization of the presynaptic marker synaptophysin (green). Scale bar is 5 μm. Images were subjected to quantitative analysis and synaptic αSyn levels were determined as the amount of signal that colocalizes with the synaptic synaptophysin marker. Automated measurements were performed in Metamorph imaging analysis software to determine synaptic αSyn and synaptophysin levels by integrated intensity or pixel area, respectively. Values represent mean +/− SEM, n = 1000 terminals (αSyn) or 18 optical fields (synaptophysin) per condition, and derived from 2–3 independent cultures. Quantitation demonstrates that ELN484228 reduces the synaptic levels of αSyn in rat hippocampal neurons.
doi:10.1371/journal.pone.0087133.g002

identify compounds with a variety of effects related to αSyn malfunction or misfolding, and we have demonstrated that indeed this screening approach yields molecules with dramatic impact on αSyn malfunction in cells.

The initial challenge was to identify small molecules that bind to αSyn despite the lack of well-defined conformational states of the protein. To surmount this challenge, we first used *in silico* structure-based fragment mapping to identify a discrete set of eight potential binding pockets identified from 100 conformations of αSyn, which are part of a structural ensemble of the protein constructed using experimental NMR data and molecular dynamics simulations [24]. We then performed an *in silico* structure-based docking screen of 33,000 compounds to identify a group of *in silico* hit compounds with potential to bind to these eight binding pockets. Using this methodology, we identified diverse small molecules that likely bind αSyn and described one of them in detail (ELN484228). Further studies will be needed to establish whether ELN484228 or other hit compounds interact with their predicted binding pockets and conformations of αSyn. It is also possible that small molecules that bind monomeric forms could interact more strongly with more ordered species, such as oligomers and fibrils. In principle, NMR techniques could be used for testing these possibilities, but as they tend to measure the bulk collection of conformational states of αSyn, they are relatively insensitive in measuring the small perturbations on the ensemble from compounds binding to minor conformations due to large averaging of chemical shifts. More sophisticated NMR methodologies such as ligand-based approaches may thus be required for these studies.

These findings and recent theoretical [19] and experimental [65] observations on the Aβ peptide associated with Alzheimer's disease suggest that IDPs in general may contain small-molecule binding sites in a subset of their conformational ensemble. Thus our fragment mapping and docking studies suggest that locally-persistent binding sites are present even within a diverse population of conformations of αSyn, similarly to the case of the Aβ peptide [19]. Binding of small molecules to such pockets could result in altering the conformational ensemble of the protein and furthermore could have effects on the dynamics and thermodynamic stability of the various conformational states of αSyn. These results suggest that a practical strategy can be developed to target small molecules to transient pockets present within the ensemble of an IDP and, that once discovered, such molecules can have a dramatic impact on reversing αSyn cellular dysfunction. In order to test if this is a viable screening strategy we tested select compounds in assays of αSyn-induced malfunction and were able to demonstrate the identification of at least one compound, ELN484228, showing protection using this screening paradigm.

The role of αSyn in vesicle dynamics in neuronal and non-neuronal cells [26,29,32,34,36] is established, and a mechanistic link between the vesicular dysregulation and αSyn toxicity has been proposed [26]. We have reported that phagocytosis, a process involving vesicular mobilization and extrusion, is impaired by αSyn overexpression in cells and mice as well as in fibroblasts and blood cells from Parkinson's patients [42]. Thus, the repair of this deficit may be a potential therapeutic approach for PD. The activity of ELN484228 in the cellular models of phagocytosis demonstrates a novel and dramatic impact of a small molecule on cellular dysfunction mediated by elevated αSyn. The corresponding protection against αSyn-induced toxicity in primary rat dopaminergic neurons indicates that ELN484228 is also protective in a model more clearly reflective of PD neurodegeneration and in addition supports the hypothetical link between αSyn impact on vesicle dynamics and toxicity proposed by Lindquist and colleagues [26].

The biological impact of ELN484228 indicates that our overall strategy of identifying αSyn binding compounds. We note, however, that we have not yet firmly proven that ELN484228 is engaging αSyn in the cells and that this process is mediating its impact. Although we have rationally selected ELN484228 to interact with αSyn, it may still have the biological activity that we report by engaging different targets. Further experiments will be needed to clarify this issue. Due to the difficulty inherent in demonstrating interaction of a ligand with a low abundance conformation of αSyn, *in vitro* as well as in the cell, this can be a challenging endeavor. The most sensitive methods for detection of interaction of compounds with the biologically critical conformations of αSyn may in fact be testing for impact on cellular function. αSyn dysfunction can arise from the ability to attain a particular minor conformation, and thus a bioassay can be much more sensitive than any bulk binding measurement. This would be particularly true if the compound is interacting with a conformation mediating the transition of αSyn to a biologically critical state such as binding to membranes. It is of note that in both the glial and neuronal cells that trafficking of αSyn to either the site of engulfment or to the synapse was associated with protective action of ELN484228, suggesting the possibility that ELN484228 may act on a conformation of αSyn or cellular process that is involved in this trafficking. To our knowledge this is the first report of a drugable small molecule that alters transport of αSyn and ameliorates the αSyn-mediated impairment of vesicular dynamics involved in phagocytosis.

We did not see protection in all models of αSyn-mediated neuronal toxicity. This result is likely to arise from the differences between the various models that we used. For example, our alternate model, in which ELN484228 did not show protection, utilizes immature rat cortical neurons, which have not yet formed synapses [63,64]. αSyn is recruited to the presynaptic terminal of mature synapses where it regulates distinct pools of synaptic vesicles and when over-expressed impairs neurotransmitter release [28,33,34]. This is a critical issue as we show that ELN484228

reduces the recruitment of αSyn to the phagocytic cup and to mature synapses, and this alteration in recruitment may underlie the beneficial mechanism of action of ELN484228 in both the phagocytosis and neuronal toxicity models. It is tempting to speculate that the impairment of vesicular function by elevated αSyn at the phagocytic cup and at synapses is reversed by the reduction in recruitment of αSyn to those sites by ELN484228. The lack of protection in immature neuronal cultures and protection in mature cultures with synapses is consistent with this hypothesis and indicates that the mechanism of αSyn toxicity in these two models could be significantly different. Alternatively, additional factors in these models could account for the differing results. Dopaminergic midbrain cells are more vulnerable in PD than cortical neurons [1] and the differing biology of αSyn in this neuronal subtype could be relevant. In addition, mutant A53T αSyn is used in the dopaminergic culture experiments versus the use of wildtype αSyn in the cortical model and likewise could be key to the differing results as the mechanism of neuronal toxicity may be different between the wild type and A53T forms of αSyn. NMR studies suggest that the A53T mutation impacts the overall conformational ensemble of αSyn [66]. Nevertheless, it is not clear whether this mutation would impact the local and tertiary conformations of the pocket to which ELN484228 is predicted to bind, as computational analysis of the A53T mutant αSyn conformational ensemble has not been performed. Protection by ELN484228 against A53T αSyn induced neurotoxicity in dopaminergic neurons suggest that ELN484228 can interact with A53T αSyn.

ELN484228 reduced synaptic levels of αSyn in neuronal cultures from both wild type rats and from transgenic mice overexpressing αSyn by not more than two-fold. Thus the amount of αSyn does not go below 50% of endogenous levels even at high doses of ELN484228. This is probably why this compound does not induce toxicity in wild type cells at efficacious doses, as there is likely to be sufficient total αSyn at the synapse for proper modulation of neurotransmission. ELN484228 is a small molecule whose central element is a benzene-sulfonamide group, a functional moiety found in several dozen approved drugs such as the antibiotics sulfanilamide, sulfapyridine and sulfadiazine [67] and thus has drug-like potential. Thus, compound ELN484228 is likely to be a good starting point for development of a small-molecule PD therapeutic designed to target αSyn.

In conclusion, we have reported that an *in silico* high-throughput structure-based docking screen resulted in the identification of several fragment-like small molecules predicted to bind to αSyn. These results support our findings that small molecule binding pockets can exist within members of the heterogeneous conformational ensemble of IDPs, including αSyn and the Aβ peptide [19] despite their overall lack of persistent structures. One of our novel drug-like binders (ELN484228) was further characterized and found to be biologically active. The impact of ELN484228 on αSyn overexpressing cells results in significant amelioration of αSyn-induced cellular perturbations including impaired vesicular dynamics, neurotoxicity, and neurite retraction, all endpoints with therapeutic potential for PD. This is the first report of a drugable small molecule that alters transport of αSyn and ameliorates the αSyn-mediated impairment of vesicular dynamics involved in phagocytosis. This finding illustrates the potential value of this molecule as both a research tool and a lead compound directed against PD and related conditions [12,68]. Further studies will be needed to verify that the engagement of αSyn in cells is mediating the protective effects of ELN484228. Overall, however, these results suggest that targeting small molecules to IDPs such as αSyn, has the potential to alter their properties in therapeutically relevant ways and provides a viable screening approach. Thus, such an integrated theoretical and experimental approach represents a promising drug discovery strategy for combating Parkinson's and other neurodegenerative diseases.

## Supporting Information

**File S1**
(DOCX)

**Figure S1 3D models of αSyn- ligand interaction from the docking calculations.** a) A representative 3D model of αSyn-spermidine interaction based on the docking calculations. *Left panel:* Entire surface representation of αSyn conformation 2; r*ight panel* spermidine binding pocket in αSyn conformation 2. Spermidine appears as magenta sticks and αSyn as surface representation or blue sticks. Black arrow illustrates a potential hydrogen bond or a salt bridge. Due to the high flexibility of spermidine and to the predominance of negatively charged side chains of the C-terminal of aSyn, spermidine has the potential to bind to many conformations of αSyn by forming charge-charge interactions. (b) A representative 3D model of αSyn-ELN484228 interaction based on the docking calculations. *Left panel*: surface representation of Conformation 2 of αSyn in complex with ELN484228. *Right panel*: close-up view of ELN484228 binding to Pocket I of Conformation 2. Black arrow illustrates a potential hydrogen bond or a salt bridge. (c) A representative model of the αSyn-ELN484217 interaction based on the docking calculations. ELN484217 is predicted to bind to the N-terminal portion of αSyn conformation 13. *Left panel*: 3D surface representation of conformation 13 of αSyn in complex with ELN484217. *Right panel*: close-up view of ELN484217 binding to the N-terminal of αSyn conformation 13 depicted in a representative 2D model of αSyn-ELN484217 showing hydrogen bonds (green arrow) and cation-aromatic interactions (green arrow with aromatic+). Residues of αSyn in the surface representation are coloured accordingly their location in primary sequence as shown at the bottom of panels on the left hand side.
(TIF)

**Figure S2 Typical profile of the results obtained after docking to a pocket in αSyn conformations.** $E_{rank}$ distribution of neutral (**a**) and charged (**c**) small molecule binders to Pocket I of Conformation 2 of αSyn. $L_{Re}$ distribution of neutral (**b**) and charged (**d**) small molecule binders to Pocket I of Conformation 2 of αSyn.
(TIF)

**Figure S3 ELN484228 did not influence the aggregation kinetics of αSyn amyloid formation.** Aggregation of αSyn in the presence of 484228 compared to the corresponding DMSO control following both (**a**) ThT signal and the fraction of soluble protein remaining in solution according to (**b**) SDS-PAGE electrophoresis. (Control1-3 is DMSO. 228b3as1-3 and 228b35s1-3 are two distinct batches of 484228). Fibrils of αSyn grown in the presence of 484228 retain the characteristic amyloid morphology (data not shown). 484228 also did not impact aggregation in a more quantitative assay in which fibril seeds were added at the initiation of the assay (data not shown).
(TIF)

**Figure S4 Compound 484228 is not cytotoxic at concentrations up to 60 μM.** Primary midbrain cultures from E17 rat embryos were incubated with or without the compound for 96 h. The data are plotted as the mean ± s.e.m. (n = 2).
(TIF)

**Figure S5 Increasing Concentrations of 484228 Does Not Reduce α-Syn mediated toxicity in cortical neurons.** Cumulative risk of death curves demonstrate that cortical neurons overexpressing α-synuclein have a significantly increased risk of toxicity (α-syn/veh (veh = vehicle) versus control/veh, hazard ratio (HR) 1.61, p value $< 0.0001$). The exposure of cortical neurons overexpressing α-synuclein to increasing concentrations of ELN484228 did not significantly reduce α-synuclein mediated toxicity (α-syn/veh versus α-syn/1uM, HR 1.09, p = 0.31; α-syn/veh versus α-syn/5uM, HR 1.1, p = 0.31; α-syn/veh versus α-syn/10uM, HR 1.03, p = 0.79). Exposure of control cells to increasing concentrations of 484228 did not cause toxicity (control/veh versus control/1uM, HR 1.02, p = 0.87; control/veh versus control/5uM, HR 0.95, p = 0.59; control/veh versus control/10uM, HR 0.95, p = 0.63). Number of neurons α-syn/veh = 287, α-syn/1uM = 237, α-syn/5uM = 258, α-syn/10uM = 206, control/veh = 276, control/1uM = 293, control/5uM = 226, control/10uM = 232, 3 independent experiments combined.
(TIF)


## Acknowledgments

The authors thank Dr. Xiao-hua Chen (Elan Pharmaceuticals) for handling the preparation of the compound DMSO stock solutions, Dr. B. Peterson (Elan Pharmaceuticals) for running analytical chemistry quality control studies on all screened compounds, Kang Hu (Elan Pharmaceuticals), and Joyce Kwan (Elan Pharmaceuticals), for their technical assistance, and Dr. David Agard for critical reading of the manuscript.


## Author Contributions

Conceived and designed the experiments: GT SG WZ CWB NC SLR MAT JR CG GS SF MB KR SC JJ KC JPA MFJ AKB TAY TPJK MV JC CMD DS LM. Performed the experiments: GT SG WZ CWB NC SLR MAT CG GS KR SC KC AKB. Analyzed the data: GT SG WZ CWB NC SLR MAT JR CG GS SF MB KR SC JJ KC JPA MFJ AKB TAY TPJK MV JC CMD DS LM. Contributed reagents/materials/analysis tools: GT SG WZ JR GS SF SC MV. Wrote the paper: GT SG WZ CWB NC JR GS KR MV CMD LM.

## References


1. Dawson TM, Dawson VL (2003) Molecular pathways of neurodegeneration in Parkinson's disease. Science 302: 819–822.
2. Spillantini MG, Schmidt ML, Lee VM, Trojanowski JQ, Jakes R, et al. (1997) Alpha-synuclein in Lewy bodies. Nature 388: 839–840.
3. Lucking CB, Durr A, Bonifati V, Vaughan J, De Michele G, et al. (2000) Association between early-onset Parkinson's disease and mutations in the parkin gene. The New England journal of medicine 342: 1560–1567.
4. Schiesling C, Kieper N, Seidel K, Kruger R (2008) Review: Familial Parkinson's disease–genetics, clinical phenotype and neuropathology in relation to the common sporadic form of the disease. Neuropathology and applied neurobiology 34: 255–271.
5. Polymeropoulos MH, Lavedan C, Leroy E, Ide SE, Dehejia A, et al. (1997) Mutation in the alpha-synuclein gene identified in families with Parkinson's disease. Science 276: 2045–2047.
6. Singleton AB, Farrer M, Johnson J, Singleton A, Hague S, et al. (2003) alpha-Synuclein locus triplication causes Parkinson's disease. Science 302: 841.
7. Fuchs J, Nilsson C, Kachergus J, Munz M, Larsson EM, et al. (2007) Phenotypic variation in a large Swedish pedigree due to SNCA duplication and triplication. Neurology 68: 916–922.
8. Saiki S, Sato S, Hattori N (2012) Molecular pathogenesis of Parkinson's disease: update. Journal of neurology, neurosurgery, and psychiatry 83: 430–436.
9. Simon-Sanchez J, Schulte C, Bras JM, Sharma M, Gibbs JR, et al. (2009) Genome-wide association study reveals genetic risk underlying Parkinson's disease. Nature genetics 41: 1308–1312.
10. Dyson HJ, Wright PE (2005) Intrinsically unstructured proteins and their functions. Nature reviews Molecular cell biology 6: 197–208.
11. Tompa P (2005) The interplay between structure and function in intrinsically unstructured proteins. FEBS letters 579: 3346–3354.
12. Chiti F, Dobson CM (2006) Protein misfolding, functional amyloid, and human disease. Annu Rev Biochem 75: 333–366.
13. Uversky VN, Dunker AK (2010) Understanding protein non-folding. Biochimica et biophysica acta 1804: 1231–1264.
14. Cohen FE, Kelly JW (2003) Therapeutic approaches to protein-misfolding diseases. Nature 426: 905–909.
15. Klabunde T, Petrassi HM, Oza VB, Raman P, Kelly JW, et al. (2000) Rational design of potent human transthyretin amyloid disease inhibitors. Nat Struct Biol 7: 312–321.
16. Bulawa CE, Connelly S, Devit M, Wang L, Weigel C, et al. (2012) Tafamidis, a potent and selective transthyretin kinetic stabilizer that inhibits the amyloid cascade. Proceedings of the National Academy of Sciences of the United States of America 109: 9629–9634.
17. Orwig SD, Tan YL, Grimster NP, Yu Z, Powers ET, et al. (2011) Binding of 3,4,5,6-tetrahydroxyazepanes to the acid-beta-glucosidase active site: implications for pharmacological chaperone design for Gaucher disease. Biochemistry 50: 10647–10657.
18. Metallo SJ (2010) Intrinsically disordered proteins are potential drug targets. Curr Opin Chem Biol 14: 481–488.
19. Zhu M, De Simone A, Schenk D, Toth G, Dobson CM, et al. (2013) Identification of small-molecule binding pockets in the soluble monomeric form of the Abeta 42 peptide. The Journal of Chemical Physics 139: 035101.
20. McInnes C (2007) Virtual screening strategies in drug discovery. Curr Opin Chem Biol 11: 494–502.
21. Vendruscolo M (2007) Determination of conformationally heterogeneous states of proteins. Curr Opin Struct Biol 17: 15–20.
22. Jensen MR, Markwick PR, Meier S, Griesinger C, Zweckstetter M, et al. (2009) Quantitative determination of the conformational properties of partially folded and intrinsically disordered proteins using NMR dipolar couplings. Structure 17: 1169–1185.
23. Bertoncini CW, Jung YS, Fernandez CO, Hoyer W, Griesinger C, et al. (2005) Release of long-range tertiary interactions potentiates aggregation of natively unstructured alpha-synuclein. Proc Natl Acad Sci U S A 102: 1430–1435.
24. Dedmon MM, Lindorff-Larsen K, Christodoulou J, Vendruscolo M, Dobson CM (2005) Mapping long-range interactions in alpha-synuclein using spin-label NMR and ensemble molecular dynamics simulations. Journal of the American Chemical Society 127: 476–477.
25. Anwar S, Peters O, Millership S, Ninkina N, Doig N, et al. (2011) Functional alterations to the nigrostriatal system in mice lacking all three members of the synuclein family. The Journal of neuroscience: the official journal of the Society for Neuroscience 31: 7264–7274.
26. Auluck PK, Caraveo G, Lindquist S (2010) alpha-Synuclein: membrane interactions and toxicity in Parkinson's disease. Annual review of cell and developmental biology 26: 211–233.
27. Ben Gedalya T, Loeb V, Israeli E, Altschuler Y, Selkoe DJ, et al. (2009) Alpha-synuclein and polyunsaturated fatty acids promote clathrin-mediated endocytosis and synaptic vesicle recycling. Traffic 10: 218–234.
28. Cabin DE, Shimazu K, Murphy D, Cole NB, Gottschalk W, et al. (2002) Synaptic vesicle depletion correlates with attenuated synaptic responses to prolonged repetitive stimulation in mice lacking alpha-synuclein. The Journal of neuroscience: the official journal of the Society for Neuroscience 22: 8797–8807.
29. Chandra S, Fornai F, Kwon HB, Yazdani U, Atasoy D, et al. (2004) Double-knockout mice for alpha- and beta-synucleins: effect on synaptic functions. Proceedings of the National Academy of Sciences of the United States of America 101: 14966–14971.
30. Cooper AA, Gitler AD, Cashikar A, Haynes CM, Hill KJ, et al. (2006) Alpha-synuclein blocks ER-Golgi traffic and Rab1 rescues neuron loss in Parkinson's models. Science 313: 324–328.
31. Gaugler MN, Genc O, Bobela W, Mohanna S, Ardah MT, et al. (2012) Nigrostriatal overabundance of alpha-synuclein leads to decreased vesicle density and deficits in dopamine release that correlate with reduced motor activity. Acta neuropathologica 123: 653–669.
32. Kim KS, Park JY, Jou I, Park SM (2010) Regulation of Weibel-Palade body exocytosis by alpha-synuclein in endothelial cells. The Journal of biological chemistry 285: 21416–21425.
33. Murphy DD, Rueter SM, Trojanowski JQ, Lee VM (2000) Synucleins are developmentally expressed, and alpha-synuclein regulates the size of the presynaptic vesicular pool in primary hippocampal neurons. The Journal of neuroscience: the official journal of the Society for Neuroscience 20: 3214–3220.
34. Nemani VM, Lu W, Berge V, Nakamura K, Onoa B, et al. (2010) Increased expression of alpha-synuclein reduces neurotransmitter release by inhibiting synaptic vesicle reclustering after endocytosis. Neuron 65: 66–79.
35. Su LJ, Auluck PK, Outeiro TF, Yeger-Lotem E, Kritzer JA, et al. (2010) Compounds from an unbiased chemical screen reverse both ER-to-Golgi trafficking defects and mitochondrial dysfunction in Parkinson's disease models. Disease models & mechanisms 3: 194–208.
36. Thayanidhi N, Helm JR, Nycz DC, Bentley M, Liang Y, et al. (2010) Alpha-synuclein delays endoplasmic reticulum (ER)-to-Golgi transport in mammalian cells by antagonizing ER/Golgi SNAREs. Molecular biology of the cell 21: 1850–1863.
37. Westphal CH, Chandra SS (2013) Monomeric synucleins generate membrane curvature. The Journal of biological chemistry 288: 1829–1840.

38. Burre J, Sharma M, Tsetsenis T, Buchman V, Etherton MR, et al. (2010) Alpha-synuclein promotes SNARE-complex assembly in vivo and in vitro. Science 329: 1663–1667.
39. Sudhof TC, Rizo J (2011) Synaptic vesicle exocytosis. Cold Spring Harbor perspectives in biology 3.
40. Palokangas H, Mulari M, Vaananen HK (1997) Endocytic pathway from the basal plasma membrane to the ruffled border membrane in bone-resorbing osteoclasts. Journal of cell science 110 (Pt 15): 1767–1780.
41. Hackam DJ, Rotstein OD, Sjolin C, Schreiber AD, Trimble WS, et al. (1998) v-SNARE-dependent secretion is required for phagocytosis. Proceedings of the National Academy of Sciences of the United States of America 95: 11691–11696.
42. Shyra J. Gardai WM, Birgitt Schüle, Michael Babcock, Sue Schoebel, Carlos Lorenzana, Jeff Alexander, Sam Kim, Heather Glick, Kathryn Hilton, J. Kent Fitzgerald, Manuel Buttini, San-San Chiou, Lisa McConlogue, John P Anderson, Dale B Schenk, Frederique Bard, J. William Langston, Ted Yednock, , and Jennifer A Johnston (2013) Elevated Alpha-Synuclein Impairs Innate Immune Cell Function and Provides a Potential Peripheral Biomarker for Parkinson's Disease. Plos One.
43. McGann M (2011) FRED pose prediction and virtual screening accuracy. Journal of chemical information and modeling 51: 578–596.
44. Kortvelyesi T, Dennis S, Silberstein M, Brown L, 3rd, Vajda S (2003) Algorithms for computational solvent mapping of proteins. Proteins 51: 340–351.
45. Landon MR, Lancia DR, Jr., Yu J, Thiel SC, Vajda S (2007) Identification of hot spots within druggable binding regions by computational solvent mapping of proteins. Journal of medicinal chemistry 50: 1231–1240.
46. Weinreb PH, Zhen W, Poon AW, Conway KA, Lansbury PT, Jr. (1996) NACP, a protein implicated in Alzheimer's disease and learning, is natively unfolded. Biochemistry 35: 13709–13715.
47. Lendel C, Bertoncini CW, Cremades N, Waudby CA, Vendruscolo M, et al. (2009) On the mechanism of nonspecific inhibitors of protein aggregation: dissecting the interactions of alpha-synuclein with Congo red and Lacmoid. Biochemistry 48: 8322–8334.
48. Hoyer W, Antony T, Cherny D, Heim G, Jovin TM, et al. (2002) Dependence of alpha-synuclein aggregate morphology on solution conditions. Journal of molecular biology 322: 383–393.
49. Uversky VN, Li J, Fink AL (2001) Evidence for a partially folded intermediate in alpha-synuclein fibril formation. J Biol Chem 276: 10737–10744.
50. Farrer M, Maraganore DM, Lockhart P, Singleton A, Lesnick TG, et al. (2001) alpha-Synuclein gene haplotypes are associated with Parkinson's disease. Human molecular genetics 10: 1847–1851.
51. Zarranz JJ, Alegre J, Gomez-Esteban JC, Lezcano E, Ros R, et al. (2004) The new mutation, E46K, of alpha-synuclein causes Parkinson and Lewy body dementia. Annals of neurology 55: 164–173.
52. Liu J, Cavalli LR, Haddad BR, Papadopoulos V (2003) Molecular cloning, genomic organization, chromosomal mapping and subcellular localization of mouse PAP7: a PBR and PKA-RIalpha associated protein. Gene 308: 1–10.
53. Gitler AD, Chesi A, Geddie ML, Strathearn KE, Hamamichi S, et al. (2009) Alpha-synuclein is part of a diverse and highly conserved interaction network that includes PARK9 and manganese toxicity. Nature genetics 41: 308–315.
54. Liu F, Nguyen JL, Hulleman JD, Li L, Rochet JC (2008) Mechanisms of DJ-1 neuroprotection in a cellular model of Parkinson's disease. Journal of neurochemistry 105: 2435–2453.
55. Anderson JP, Walker DE, Goldstein JM, de Laat R, Banducci K, et al. (2006) Phosphorylation of Ser-129 is the dominant pathological modification of alpha-synuclein in familial and sporadic Lewy body disease. The Journal of biological chemistry 281: 29739–29752.
56. Arrasate M, Mitra S, Schweitzer ES, Segal MR, Finkbeiner S (2004) Inclusion body formation reduces levels of mutant huntingtin and the risk of neuronal death. Nature 431: 805–810.
57. Andersen PG (1982) Cox's regression model for counting processes, a large sample study. Annals of Statistics. 10: 1100–1120.
58. Allison JR, Varnai P, Dobson CM, Vendruscolo M (2009) Determination of the free energy landscape of alpha-synuclein using spin label nuclear magnetic resonance measurements. J Am Chem Soc 131: 18314–18326.
59. Salmon L, Nodet G, Ozenne V, Yin G, Jensen MR, et al. (2010) NMR characterization of long-range order in intrinsically disordered proteins. J Am Chem Soc 132: 8407–8418.
60. Clackson T, Wells JA (1995) A hot spot of binding energy in a hormone-receptor interface. Science 267: 383–386.
61. Conway KA, Lee SJ, Rochet JC, Ding TT, Williamson RE, et al. (2000) Acceleration of oligomerization, not fibrillization, is a shared property of both alpha-synuclein mutations linked to early-onset Parkinson's disease: implications for pathogenesis and therapy. Proceedings of the National Academy of Sciences of the United States of America 97: 571–576.
62. Outeiro TF, Kontopoulos E, Altmann SM, Kufareva I, Strathearn KE, et al. (2007) Sirtuin 2 inhibitors rescue alpha-synuclein-mediated toxicity in models of Parkinson's disease. Science 317: 516–519.
63. Nakamura K, Nemani VM, Azarbal F, Skibinski G, Levy JM, et al. (2011) Direct membrane association drives mitochondrial fission by the Parkinson disease-associated protein alpha-synuclein. The Journal of biological chemistry 286: 20710–20726.
64. Sharma P, Ando DM, Daub A, Kaye JA, Finkbeiner S (2012) High-throughput screening in primary neurons. Methods in enzymology 506: 331–360.
65. Chen J, Armstrong AH, Koehler AN, Hecht MH (2010) Small molecule microarrays enable the discovery of compounds that bind the Alzheimer's Abeta peptide and reduce its cytotoxicity. J Am Chem Soc 132: 17015–17022.
66. Bertoncini CW, Fernandez CO, Griesinger C, Jovin TM, Zweckstetter M (2005) Familial mutants of alpha-synuclein with increased neurotoxicity have a destabilized conformation. The Journal of biological chemistry 280: 30649–30652.
67. Wishart DS, Knox C, Guo AC, Cheng D, Shrivastava S, et al. (2008) DrugBank: a knowledgebase for drugs, drug actions and drug targets. Nucleic Acids Res 36: D901–906.
68. Dobson CM (2003) Protein folding and misfolding. Nature 426: 884–890.
